# Variations in the practice of molecular radiotherapy and implementation of dosimetry: results from a European survey

**DOI:** 10.1186/s40658-017-0193-4

**Published:** 2017-12-04

**Authors:** Katarina Sjögreen Gleisner, Emiliano Spezi, Pavel Solny, Pablo Minguez Gabina, Francesco Cicone, Caroline Stokke, Carlo Chiesa, Maria Paphiti, Boudewijn Brans, Mattias Sandström, Jill Tipping, Mark Konijnenberg, Glenn Flux

**Affiliations:** 1 Department of Medical Radiation Physics, Clinical Sciences Lund, Lund University, Lund, Sweden; 2 School of Engineering, Cardiff University, Cardiff, UK; 3 Department of Nuclear Medicine and Endocrinology, Motol University Hospital, 2nd Faculty of Medicine, Charles University, Prague, Czech Republic; 4 Department of Medical Physics and Radiation Protection, Gurutzeta/Cruces University Hospital, Barakaldo, Spain; 5 Nuclear Medicine, Sant’Andrea Hospital, Department of Surgical and Medical Sciences and Translational Medicine, Sapienza University of Rome, Rome, Italy; 6 Department of Diagnostic Physics, Oslo University Hospital, Oslo, Norway; 7 Nuclear Medicine Division, Foundation IRCCS Istituto Nazionale Tumori, Milan, Italy; 8 Department of Medical Physics, Pammakaristos Hospital, Athens, Greece; 9 Department of Nuclear Medicine and PET Center, University Hospital, Ghent, Belgium; 10 Department of Surgical Sciences, Radiology, Uppsala University Hospital, Uppsala, Sweden; 11 The Christie NHS Foundation Trust, Nuclear Medicine, Manchester, UK; 12 Department of Nuclear Medicine, Erasmus MC, Rotterdam, The Netherlands; 13 Joint Department of Physics, Royal Marsden Hospital and Institute of Cancer Research, Sutton, UK

**Keywords:** Molecular radiotherapy, Radionuclide therapy, Radiopharmaceutical therapy, European survey, Dosimetry

## Abstract

**Background:**

Currently, the implementation of dosimetry in molecular radiotherapy (MRT) is not well investigated, and in view of the Council Directive (2013/59/Euratom), there is a need to understand the current availability of dosimetry-based MRT in clinical practice and research studies. The aim of this study was to assess the current practice of MRT and dosimetry across European countries.

**Methods:**

An electronic questionnaire was distributed to European countries. This addressed 18 explicitly considered therapies, and for each therapy, a similar set of questions were included. Questions covered the number of patients and treatments during 2015, involvement of medical specialties and medical physicists, implementation of absorbed dose planning, post-therapy imaging and dosimetry, and the basis of therapy prescription.

**Results:**

Responses were obtained from 26 countries and 208 hospitals, administering in total 42,853 treatments. The most common therapies were ^131^I-NaI for benign thyroid diseases and thyroid ablation of adults. The involvement of a medical physicist (mean over all 18 therapies) was reported to be either minority or never by 32% of the responders. The percentage of responders that reported that dosimetry was included on an always/majority basis differed between the therapies and showed a median value of 36%. The highest percentages were obtained for ^177^Lu-PSMA therapy (100%), ^90^Y microspheres of glass (84%) and resin (82%), ^131^I-mIBG for neuroblastoma (59%), and ^131^I-NaI for benign thyroid diseases (54%). The majority of therapies were prescribed based on fixed-activity protocols. The highest number of absorbed-dose based prescriptions were reported for ^90^Y microsphere treatments in the liver (64% and 96% of responses for resin and glass, respectively), ^131^I-NaI treatment of benign thyroid diseases (38% of responses), and for ^131^I-mIBG treatment of neuroblastoma (18% of responses).

**Conclusions:**

There is a wide variation in MRT practice across Europe and for different therapies, including the extent of medical-physicist involvement and the implementation of dosimetry-guided treatments.

**Electronic supplementary material:**

The online version of this article (10.1186/s40658-017-0193-4) contains supplementary material, which is available to authorized users.

## Background

Molecular radiotherapy (MRT) refers to the use of internally distributed, unsealed radioactive substances for the treatment of benign and malignant diseases. These radioactive substances, namely radiopharmaceuticals, represent the combination of an unstable radionuclide with an active or pharmacologically inert molecule. The energy released by the radionuclide at decay determines or boosts the therapeutic effect of the vector molecule. Initial reports of MRT in humans date back to the period between 1938 and 1939, when several patients suffering from chronic myeloid and lymphoid leukemia were treated with repeated oral administrations of ^32^P sodium phosphate, which accumulates in blood cells [1].

Therapeutic radiopharmaceuticals are often administered intravenously, although oral, intra-cavity, and intra-arterial administrations are also employed. They are designed to accumulate in a target tissue and, if administered systemically, are able to simultaneously treat disseminated disease which represents a clear advantage over other more localized radiotherapy modalities. Suitable radionuclides for therapy emit particle radiation, such as electrons from β^−^-decay or alpha particles, and have a longer physical half-life compared to radionuclides used for diagnostic purposes. In addition, most therapeutic radionuclides have associated nuclear emissions (positrons or gamma rays) or yield bremsstrahlung during the slowing-down of electrons. The resulting photon radiation can be detected by external probes or imaged by scintillation cameras, either in planar mode or employing single-photon (SPECT) or positron emission (PET) tomography, offering the unique possibility of live imaging and measurement of the distribution of the therapeutic agent in vivo. Based on the results of such measurements, the distribution of activity and absorbed dose can be individually determined for the relevant organs and target tissues.

For radiotherapy modalities such as external beam radiotherapy or brachytherapy, it would not be acceptable to treat patients without an accurate therapy planning, including determination of the absorbed doses delivered to target tissues and to organs at risk. For MRT, however, personalized dosimetry-based treatments have been the slowest to develop among the existing radiotherapy modalities. A forthcoming Council Directive (2013/59/Euratom) [2] mandates the use of dosimetry-based treatment planning and verification of the absorbed doses delivered. In Chapter VII, Medical Exposures, Article 56, Optimisation, it is stated that:

For all medical exposure of patients for radiotherapeutic purposes, exposures of target volumes shall be individually planned and their delivery appropriately verified taking into account that doses to non-target volumes and tissues shall be as low as reasonably achievable and consistent with the intended radiotherapeutic purpose of the exposure.

According to the same Directive (Chapter II, Definitions, Article 4, Definitions):

“radiotherapeutic” means pertaining to radiotherapy, including nuclear medicine for therapeutic purposes.

This directive is to be implemented in national legislations by 6 February 2018.

The EANM Internal Dosimetry Task Force (IDTF) was formed to address aspects of the 2013/59/ Euratom Directive specifically concerned with dosimetry for MRT. This task force has representation from Belgium, Czech Republic, Germany, Greece, Italy, The Netherlands, Norway, Spain, Sweden, Switzerland, Turkey, and the UK. To the best of the knowledge of this group, the current practice of MRT and implementation of dosimetry in clinical routine and research is poorly investigated. In 1999, results from a questionnaire distributed among EANM representatives were reported [3], which mainly addressed the practice of MRT from a clinical and medical point of view. A wide variation in therapy practice across European centers was revealed, and the authors concluded that there was a need of more uniform guidelines and legislations. Since then, the role of MRT has expanded, as more radiopharmaceuticals and treatments have been introduced. Hence, the motivation has grown to undertake a survey with the aim of defining a clear picture of the practice and variation in MRT across Europe and the current readiness of compliance with the 2013/59/Euratom Directive.

## Methods

The survey was developed by the IDTF and focused on treatments given during the year 2015. It was written in English and implemented in a web-based questionnaire. The survey was structured so that it could be completed from start to end or by navigating to particular therapies by choice. It could also be saved and continued at a later time. An introductory page explained the survey aim, gave an overview of the included therapies, and contained an option to accept or decline that results, if anonymized but traceable to the country, would be made public.

### Survey on therapies

The electronic questionnaire was constructed as one page per therapy. There were 18 explicitly considered therapies, as listed in Table 1. In addition to these, there were three pages for “Therapy using alpha emitting radionuclides other than ^223^Ra” and three pages for “Therapy using other radiopharmaceutical.”

**Table 1:** Therapy types included in the survey, each on a separate page. Therapies A–R were explicitly asked for, while S–U and V–X were included as complement

Annotation	Therapy type
A	^131^I-NaI for benign thyroid diseases
B	^131^I-NaI for thyroid remnant ablation of adults
C	^131^I-NaI for thyroid remnant ablation of children and young adults
D	^131^I-NaI for thyroid cancer therapy for adults
E	^131^I-NaI for thyroid cancer therapy for children and young adults
F	^131^I-mIBG for neuroblastoma
G	^131^I-mIBG for adult neuroendocrine tumors
H	^177^Lu-somatostatin analogues for neuroendocrine tumors
I	^90^Y-somatostatin analogues for neuroendocrine tumors
J	^177^Lu-PSMA therapy of castration resistant prostate cancer
K	^90^Y resin microspheres (SIR-Spheres®) for intra-arterial treatments in the liver
L	^90^Y glass microspheres (TheraSphere®) for intra-arterial treatments in the liver
M	Radiation synovectomy using ^90^Y-, ^186^Re-, or ^169^Er-colloids
N	^153^Sm-EDTMP (Quadramet®) for bone metastases
O	^89^SrCl_2_ (Metastron®) for bone metastases
P	^223^RaCl_2_ (Xofigo®) for bone metastases
Q	^32^P sodium-phosphate (Na_3_^32^PO_4_) for myeloproliferative disease
R	^90^Y-ibritumomab-tiuxetan (Zevalin®) for B-cell lymphoma
S–U	Therapy using alpha emitting radionuclides other than ^223^Ra
V–X	Therapy using other radiopharmaceutical

Each therapy page consisted of three sections. The first section was common for all therapies and addressed (a) the number of patients and treatments performed in 2015, (b) the medical specialty owning the license to administer treatment, (c) whether a medical physicist was involved in each treatment, (d) if the absorbed dose was individually planned, (e) whether post-therapy imaging was conducted, and (f) if post-therapy dosimetry was performed.

The second section concerned the basis of prescription for the particular therapy with 12 possible options (Table 2). The typical options included were 1–6 and 12, and multiple replies could be given. Alternative 7 was included for treatments using ^32^P sodium-phosphate or ^90^Y-ibritumomab-tiuxetan, whereas alternatives 8–11 were included for ^90^Y microsphere therapies. The subsequent questions specified the relevant responses, asking for the amount and unit of the prescription.

**Table 2:** Reply alternatives for the question “What basis of prescription do you typically use?” Therapy types are denoted A–R, as listed in Table 1. Alternatives denoted “All” implies that all therapies (A–R) included this option

No.	Included in questions	Prescription basis
1	All	Fixed activity
2	All	Fixed activity adjusted based on diagnosis, stage, or other clinical factors
3	A–J, M–R	Fixed activity calculated per body surface area or patient weight
4	A–J, N–R	Individually calculated activity to give a prescribed absorbed dose to whole body
5	A–J, N–R	Individually calculated activity to give a prescribed absorbed dose to organ at risk
6	A–J, M–R	Individually calculated activity to give a prescribed absorbed dose to target tissue
7	Q, R	Stepwise escalation of activity based on patient response
8	K, L	Body-Surface-Area (BSA) method with reductions
9	K, L	Individually calculated activity based on prescribed absorbed dose to the target volume (lobe or segment) to be treated
10	K, L	Individually calculated activity based on ^99m^Tc MAA SPECT to give a prescribed absorbed dose to non-tumoral liver tissue
11	K, L	Individually calculated activity based on 99mTc MAA SPECT to give a prescribed absorbed dose to tumor
12	All	Other prescription

The third section concerned other therapy-specific questions. These included (a) whether or not the therapy is generally repeated, (b) the typical repetition frequency, and (c) for which reason a planned sequence of therapies would be discontinued. The response types in this section were given as drop-down lists, multiple choice questions, or free-text comments.

The final page concerned more general questions, on (a) the satisfaction of the current implementation of patient-specific dosimetry in the center, (b) which factors limit its implementation, and (c) whether any research was conducted. The time required for responding was largely determined by the number of therapies that a particular center was providing and whether data on patient statistics over 2015 had been compiled prior to responding. The survey was launched June 6, 2016, by the EANM Office and via the official route by the EANM National delegates in 40 different countries.^1^ In July 2016, kind reminders were sent to the delegates and the survey was also distributed via other channels such as national networks, societies of medical physicists and nuclear medicine practitioners, as well as personal contacts. The survey was closed on September 16, 2016.

### Data analysis

All entries in the web-database were exported to a file and curated manually. This process included merging records and removing duplicate entries and inconsistent data. The spreadsheet was then imported, processed, and analyzed in the Interactive Data Language (IDL, Harris Geospatial Solutions, Broomfield, CO).

## Results

**Fig. 1:**
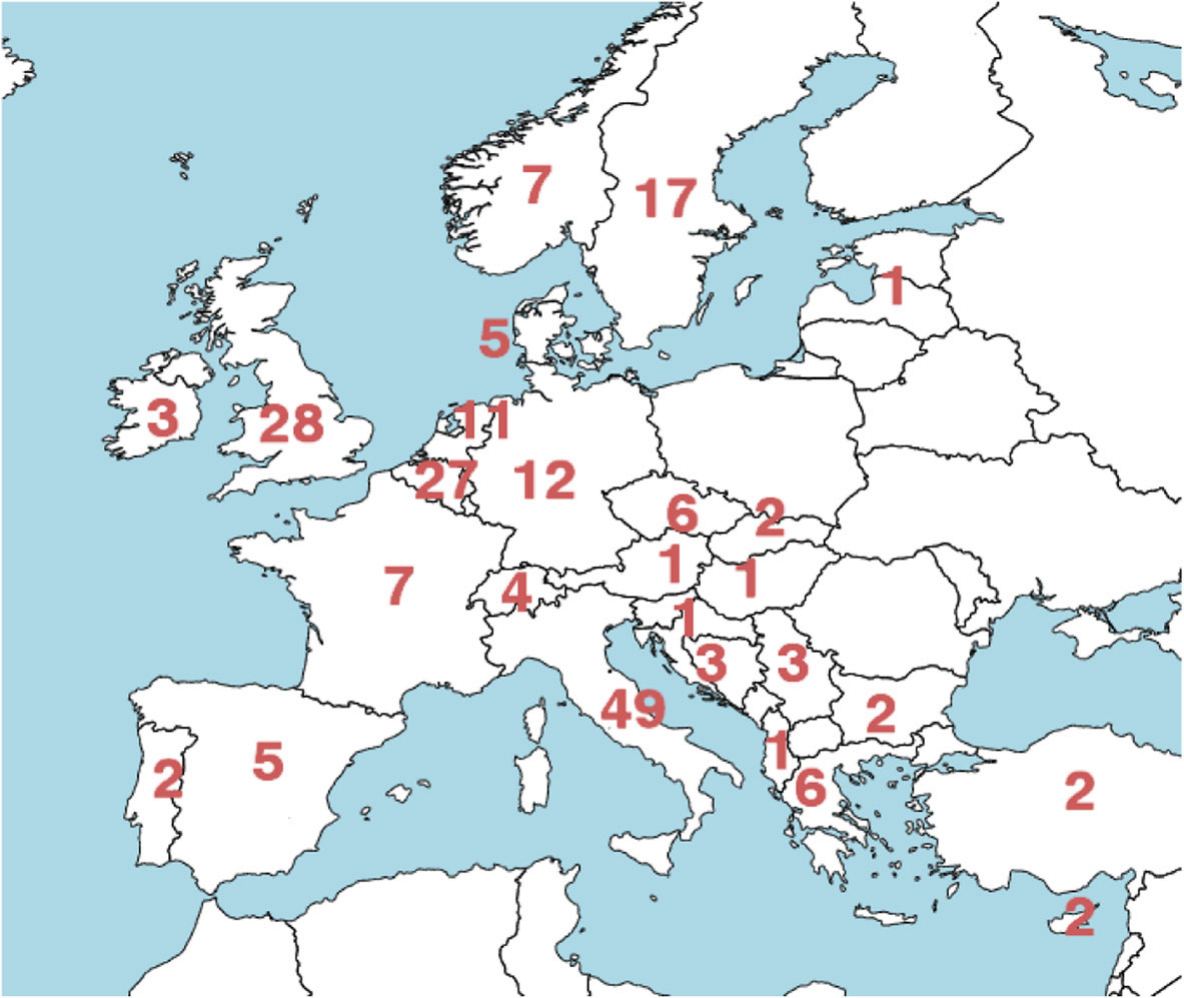
Number of responding centers in different European countries. No responses were received from countries without numbers

The total number of responders was 208, geographically distributed over 26 countries in Europe, as shown in Fig. 1.

### Number of patients and number of treatments

Table 3 shows the number of treatments per center in different countries, as well as the total number of treatments. In total, 34,838 patients were reported to be treated in the 208 centers, and as some therapies were administered multiple times, the total number of treatments was slightly higher, and was 42,853. Table 3 also lists the percentage of treatments that represented the different procedures. Therapies involving ^131^I-NaI (A-E) represented 71% of the total number of treatments given and 84% of the patients treated in total (data not shown). Of the total number of treatments, 11% were delivered using ^177^Lu/^90^Y-somatostatin analogues (peptide-receptor radionuclide therapy, PRRT) (H–I) or ^177^Lu-PSMA (J), 10% using ^223^RaCl_2_ (P), 3.7% with ^90^Y microspheres (K, L) and 2.2% with radiation synovectomy (M). As seen in Table 3, the therapies in most wide-spread use were those involving ^131^I-NaI, in particular for treatment of benign thyroid diseases and thyroid ablation of adults. Certain therapies such as ^177^Lu-PSMA (J), ^90^Y PRRT (I), ^32^P sodium-phosphate (Q), and ^90^Y-ibritumomab-tiuxetan (R) were administered only in a few countries.

**Table 3:** The number of reported treatments in a country divided by the number of responding centers that administered the particular treatment in that country. On bottom is shown the number of treatments in all countries and all responding centers, as well as the percent of the total number of treatments that represented a particular type of therapy. Therapies are numbered A–R as listed in Table 1

Country	A	B	C	D	E	F	G	H	I	J	K	L	M	N	O	P	Q	R
Albania	25	35	1.0	0	1.0	0	0	0	0	0	0	0	0	0	0	0	0	0
Austria	0	0	0	0	0	4.0	0	47	0	11	25	2.0	25	0	0	200	0	0
Belgium	26	24	1.8	14	1.0	1.5	1.3	65	0	0	16	9.0	5.0	2.1	3.0	26	0	0
Bosnia and Herzegovina	58	16	0	8.0	0	0	0	0	0	0	0	0	0	0	0	0	1.0	0
Bulgaria	38	119	1.0	112	2.0	0	0	0	0	0	0	0	0	0	0	0	0	0
Croatia	75	160	8.0	24	2.0	11	0	0	0	0	0	0	0	0	0	0	0	0
Cyprus	3.0	100	1.0	9.0	0	0	0	0	0	0	0	0	0	0	0	0	0	0
Czech Republic	39	239	9.0	146	6.0	8.0	19	0	0	0	0	0	42	16	1.5	5.0	0	0
Denmark	159	102	0	111	0	0	1.0	3.0	57	0	5.0	0	0	0	0	0	0	0
France	20	62	0	44	0	0	0	20	0	0	4.0	14	20	2.0	1.0	0	0	0
Germany	255	69	4.0	56	5.5	6.0	2.5	111	38	59	36	12	46	7.0	0	60	0	3.0
Greece	17	247	2.0	94	0	0	3.0	4.3	9.0	0	10	0	0	8.8	3.0	0	0	0
Hungary	187	44	0	5.0	0	0	0	0	0	0	0	0	0	4.0	0.0	0	0	0
Ireland	16	17	0	6.0	0	0	0	0	0	0	6.0	0	2.0	0	0	5.5	0	0
Italy	96	253	7.9	82	12	2.3	2.5	160	333	0	27	14	27	6.2	12	31	0	3.2
Latvia	198	118	0	104	0	1.0	1.0	0	0	0	0	0	0	0	0	0	0	0
Netherlands	72	15	2.0	9.2	0	6.0	4.0	296	0	0	28	50	3.3	7.8	2.7	22	2.3	0
Norway	47	27	0	67	0	2.0	2.0	0	0	0	0	0	3.0	2.5	0	58	0	0
Portugal	36	0	0	0	0	0	0	0	0	0	0	0	0	0	0	0	0	0
Serbia	95	124	4.0	53	1.5	2.0	6.0	32	36	0	0	0	0	0	0	0	0	0
Slovakia	31	458	4.0	288	4.0	0	0	0	0	0	0	0	6.0	0	1.0	23	0	0
Spain	181	114	3.5	65	1.0	2.0	2.5	3.0	0	0	6.0	6.0	22	1.3	0	26	2.0	1.0
Sweden	92	46	7.0	15	1.5	1.5	2.5	168	0	0	14	9.0	0	2.7	1.0	56	8.7	0
Switzerland	37	117	1.5	33	1.0	0	0	11	0	0	109	15	8.0	3.5	0	51	0	1.0
Turkey	20	50	0	16	0	0	0	0	0	0	0	0	0	5.0	0	0	0	0
United Kingdom	86	41	2.8	15	4.7	7.0	3.9	29	20	0	20	5.7	8.0	4.0	2.0	122	5.0	1.0
Number of treatments in all countries and all responding centers																		
	14,426	11,096	185	4706	159	113	102	2417	1926	363	1188	394	958	253	42	4343	138	44
Percent of total number of treatments																		
	34	26	0.43	11	0.37	0.26	0.24	5.6	4.5	0.85	2.8	0.92	2.2	0.59	0.10	10	0.32	0.10

### License to administer treatment

For the question “Which medical specialty owns the license to administer treatment?”, several, non-exclusive replies could be chosen among the alternatives Nuclear Medicine, Medical Oncology, Radiation Oncology, Endocrinology, Interventional Radiology, or Other. Results showed a pronounced weighting towards Nuclear Medicine, for which the average frequency for all treatments was 83%. The average frequencies for the other alternatives were for Medical Oncology (8.5%), Radiation Oncology (6.0%), Endocrinology (1.2%), and Interventional Radiology (1.0%). No center replied Other. The replies were similar for most treatments, exceptions being ^90^Y microspheres for which Interventional Radiology was higher at 12% and 6% for resin and glass, respectively, and ^32^P sodium-phosphate where the respective frequencies were for Nuclear Medicine (59%), Medical Oncology (22%), and Radiation Oncology (19%). Seventeen countries responded 100% Nuclear Medicine, while the percentage of Nuclear Medicine for the remaining countries were Belgium (96%), Bulgaria (42%), Denmark (92%), Ireland (58%), Italy (94%), Norway (84%), Sweden (30%), Turkey (14%), and UK (37%), as calculated over all therapies.

### Imaging, dosimetry, and involvement of medical physicist

For these set of questions, one exclusive reply consisting of Always, Majority, Minority, or Never could be given. The numbers were expressed as frequencies for each therapy, calculated as the number of times a particular response was given divided by the total number of responses for that therapy. Analysis was also made by combining the data into two categories, namely Always/Majority versus Minority/Never, by counting the centers that gave one of the two replies and dividing by the total number of responses for that therapy.

**Fig. 2:**
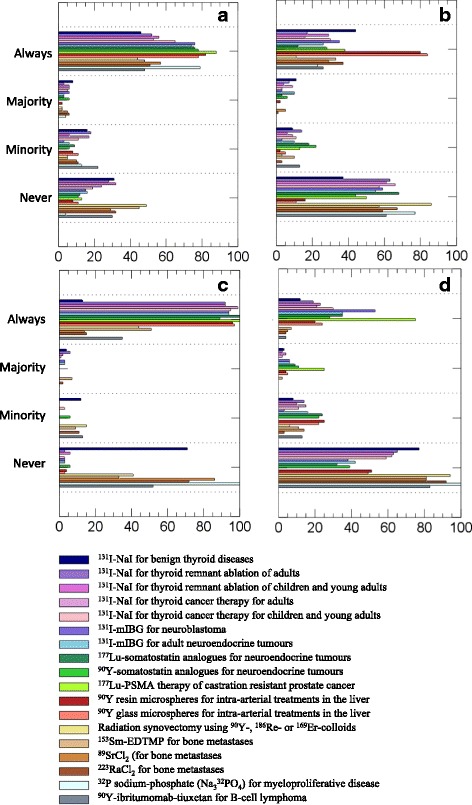
Replies for the questions **a** “Is a medical physicist involved in each treatment?”, **b** “Is the absorbed dose individually planned for each patient?”, **c** “Is post-therapy imaging performed?”, and **d** “Is post-therapy dosimetry performed?”. Numbers on the horizontal axis represent the percentage of the total number of responses for a particular therapy that gave the indicated reply

Figure 2a shows the responses on the question “Is a medical physicist involved in each treatment?”. The mean frequencies for all treatments together were for Always (63.9%), Majority (3.7%), Minority (9.7%), and Never (22.7%). When combining the data in two categories, the frequencies were thus 67.6% and 32.4%, for Always/Majority and Minority/Never, respectively. The median of the Always/Majority frequencies was 66% (min 45%, max 88%). The Always/Majority frequency was 50% or higher for all therapies except radiation synovectomy (45%) and ^90^Y-ibritumomab-tiuxetan (48%). Treatments for which the Always/Majority frequency was higher than 75% were ^177^Lu-PSMA (88%), ^90^Y PRRT (83%), ^32^P sodium-phosphate (83%), ^131^I-mIBG for neuroblastoma (82%), ^177^Lu PRRT (79%), ^131^I-mIBG for adult neuroendocrine tumors (77%), and ^90^Y microspheres of resin (84%) and glass (78%). The highest frequencies were notably obtained for therapies with a limited geographical spread (^177^Lu-PSMA: 2 countries; ^90^Y PPRT: 6 countries; and ^32^P sodium-phosphate: 4 countries).

Figure 2b shows the responses to the question “Is the absorbed dose individually planned for each patient?”. The mean frequencies for all treatments together were for Always (33.1%), Majority (3.5%), Minority (8.4%), and Never (55.1%). For the two categories Always/Majority and Minority/Never, the frequencies were thus 36.5% versus 63.5%. The median of the Always/Majority frequencies was 33% (min 11%, max 84%). The highest frequencies were obtained for ^90^Y microspheres, for which 82% (resin) and 84% (glass) of the centers responded Always/Majority, and ^131^I-NaI for benign thyroid diseases, where this frequency was 54%.

Figure 2c shows the response frequencies to the question “Is post-therapy imaging performed?”. The mean frequencies for all treatments together were for Always (67.7%), Majority (1.6%), Minority (3.7%), and Never (27.0%). For the two categories Always/Majority and Minority/Never, the respective frequencies were thus 69.3% and 30.7%. The median of the Always/Majority frequencies was 94% (min 0%, max 100%). A group of therapies had more than 50% of the centers respond Minority/Never (^131^I-NaI for benign thyroid diseases, radiation synovectomy, ^89^SrCl_2_, ^223^RaCl_2_, ^32^P sodium-phosphate, and ^90^Y-ibritumomab-tiuxetan). ^153^Sm-EDTMP for bone metastases was close to 50%, whereas for the other therapies post-therapy imaging was being conducted on an Always/Majority basis.

Figure 2d shows the response frequencies to the question “Is post-therapy dosimetry performed?” The mean frequencies for all treatments together were for Always (22.0%), Majority (4.4%), Minority (12.0%), and Never (61.6%). For the two categories Always/Majority and Minority/Never, the respective frequencies were thus 26.4% and 73.6%. The median of the Always/Majority frequencies was 23% (min 0%, max 100%). For two therapies more than 50% of the centers responded Always/Majority, ^177^Lu-PSMA (100%) and ^131^I-mIBG for neuroblastoma (59%). For PRRT using ^90^Y or ^177^Lu, as well as ^131^I-mIBG for adult neuroendocrine tumors, approximately 40% of the centers responded Always/Majority. For the other therapies, the responses on post-therapy dosimetry were mainly Minority/Never.

To summarize, the highest frequencies of any dosimetry, i.e., either as part of treatment planning or post-therapy and on an Always/Majority basis (Fig. 2b, d), were for ^177^Lu-PSMA therapy (100%), ^90^Y microspheres of glass (84%) and resin (82%), ^131^I-mIBG for neuroblastoma (59%), and ^131^I-NaI for benign thyroid diseases (54%). When calculated over all therapies, the percentage of responders that reported that dosimetry was included on an Always/Majority basis was obtained to a median of 36%.

### Activity prescription

For this set of questions, several, non-exclusive replies could be given, where the alternatives for the different therapies are listed in Table 2. The frequencies were calculated as the occurrence of a particular reply in relation to the total number of replies. Table 4 summarizes the most typically stated prescribed amounts, where for many therapies, a high diversity was obtained among the different prescription alternatives. Occasional outliers, likely caused by typographic errors, were disregarded for this summary.

**Table 4:** The most commonly stated prescribed amounts for the different therapies. Therapy notations follow Table 1, whereas prescription alternatives 1–6 refer to Table 2. The prescribed amounts are given either as median, 10th and 90th percentiles, or as median, minimum, and maximum, depending on the replies obtained. The percentiles are used to better accommodate some occasional outlier values

Therapy	Specification	Prescription alternative according to Table 2						
		Alt. 1 or 2 (unit MBq)			Alt.3, 4, 5, or 6 (unit as given)			
		Median	10th	90th	Median	10th	90th	
A	Toxic or nontoxic multinodular goiter	550	370	740	150	120	300	Gy to target
	Autonomous nodules	500	370	770	300	200	400	Gy to target
	Graves’ disease	400	222	600	200	100	300	Gy to target
B	Lowest prescribed amount	1110	1100	3490				
	Highest prescribed amount	4440	3540	7400				
C	Lowest prescribed amount	1110	1000	3700				
	Highest prescribed amount	3700	1554	6290				
D	Lowest prescribed amount	4000	1111	6920				
	Highest prescribed amount	7400	5500	10,000				
E	Lowest prescribed amount	3700	1100	5500				
	Highest prescribed amount	5525	3700	7360				
M	90-Y	185	185	220				
	169-Er	37	17	39				
	186-Re	77	73	161				
N		2545	–	–	37 MBq/kg			
O		150	148	185				
P		–	–	–	0.05–0.055 MBq/kg			
Q		185	–	–	3.7–3.9 MBq/kg or 74–111 MBq/m2			
R		–	–	–	11–15 MBq/kg (median 14.8 MBq/kg)			
		Median	Min	Max				
F		7400	2700	9250	444 MBq/kg, or 2 or 4 Gy to whole body			
G		7400	3700	14,800	444 MBq/kg, or 2 to whole body			
H		7400	4000	9175	23 or 29 Gy to kidneys			
I		2600	1480	5550	1850–2500 MBq/m2, or 23 Gy to kidneys			
J		6000	3500	7400	23 Gy to kidneys			

**Fig. 3:**
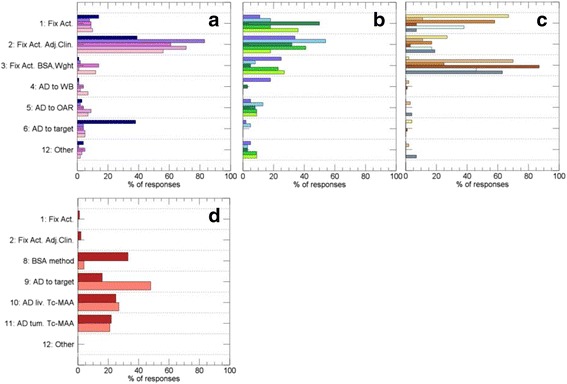
Replies for the question “What basis of prescription do you typically use?”. **a** Therapies using ^131^I-NaI (A–E in Table 1). **b** Results for ^131^I-mIBG, ^177^Lu /^90^Y PRRT, and ^177^Lu-PSMA (F–J in Table 1). **c** Therapies including radiation synovectomy, ^153^Sm-EDTMP, ^89^SrCl_2_, and ^223^RaCl_2_, ^32^P sodium-phosphate, and ^90^Y-ibritumomab-tiuxetan (M–R in Table 1). **d** ^90^Y microspheres of resin or glass (K and L in Table 1). Numbers on the horizontal axis represent the percentage of the total number of responses for a particular therapy that gave the indicated reply. The vertical axis alternatives are numbered as listed in Table 1, where explanations of the abbreviations are given. Bar colors correspond to those in Fig. 2

For therapies involving ^131^I-NaI (Fig. 3a, A–E in Table 4), the majority of the responders stated that they use different kinds of fixed-activity schemas, mainly adjusted based on the diagnosis, stage, or other clinical factors. For treatments (B–E), responses 1–3 were given on average for 84% of the replies, while for ^131^I-NaI for benign thyroid diseases (A) these responses were given in 54% of the replies, and 71 out of the 188 different responses (38%) stated that the activity was individually calculated to give a prescribed absorbed dose to target tissue.

Likewise, for therapies shown in Fig. 3b (F–J in Table 4) responses 1–3 were most frequent, thus stating that fixed activities were being used, possibly adjusted based on the diagnosis, stage or other clinical factors, or patient weight. For ^131^I-mIBG treatment of neuroblastoma (F), 8 out of 44 responses (18%) stated prescriptions based on the whole-body absorbed dose, with a limit of either 2 or 4 Gy for two subsequent administrations. Prescriptions based on the absorbed dose to organ at risk were reported for ^131^I-mIBG treatment of adult neuroendocrine tumors in 5/39 of responses (13%), for ^177^Lu PRRT in 3/38 (8%), and for ^90^Y PRRT in 2/22 (9%).

For therapies shown in Fig. 3c, including radiation synovectomy, the bone seekers ^153^Sm-EDTMP, ^89^SrCl_2_, and ^223^RaCl_2_, ^32^P sodium-phosphate, and ^90^Y-ibritumomab-tiuxetan, prescriptions listed as alternatives responses 1, 2, 3, 7 in Table 2, represented 96% of the replies, calculated as an average of the therapies. Prescriptions for these therapies were thus approximately only based on fixed-activity schemas, with adjustments made.

For ^90^Y-microsphere treatments (K, L), shown in Fig. 3d, the replies differed from above. In this case, the most common responses were prescriptions based on the absorbed dose, either to the target tissue (lobe or segment), or to non-tumor liver tissue or tumor based on ^99m^Tc-MAA SPECT (Table 2, alternatives 9–11). For resin microspheres, the frequencies were 14/85 (15%) for alternative 9 in Table 2, 21/85 (25%) for alternative 10, and 19/85 (22%) for alternative 11. For glass microspheres, the corresponding frequencies were 23/48 (48%) for alternative 9, 13/48 (27%) for alternative 10, and 10/48 (21%) for alternative 11. Thus, absorbed-dose based prescriptions were reported in 64% and 96% of responses for resin and glass, respectively. There was a difference between resin and glass microspheres in that the body-surface area method was stated in 28/85 (33%) of the responses for resin, whereas the corresponding number for glass microspheres was 2/48 (4%).

### User satisfaction

For the question “Are you satisfied with the current implementation of patient-specific dosimetry in your centre?”, 45% of the responders answered Yes and 55% answered No. For the question “Which factors limit the implementation of patient-specific dosimetry in your centre?”, the reply alternatives were non-exclusive. The listed alternatives and percentage responses were “Shortage of knowledge and know-how” (12%), “Shortage of medical physicists working in nuclear medicine” (20%), “Shortage of other staff” (13%), “Limited access to scanner or other equipment needed” (17%), “Limited access to dedicated software” (16%), “No legislative requirement to perform dosimetry” (12%), and “Other” (10%)*.*

## Discussion

To the best of our knowledge, this is the first extensive survey focused on the implementation of MRT and dosimetry. As for surveys in general, results can only be considered as a sample of an underlying true population. The extent to which the results are representative of general practice is dependent on the number of responders, their geographic spread, how well the questions were understood, and whether they could be responded to in a reasonable amount of time. Another factor that may influence the representativeness of our results is that the survey was answered on a voluntary basis and there is the possible risk that centers involved in dosimetry could be more likely to respond. Moreover, the professional role of the individual responder, e.g., physician, medical physicist, or technologist, was not asked for. For this survey, 208 responses were obtained, which we consider to be good. However, results from this survey should be regarded as a starting point with results that provide a snap-shot of the current situation.

### Total number of centers administering MRT in Europe

To our knowledge, there is no current record of the number of MRTs performed in Europe. The International Atomic Energy Agency (IAEA) keeps a database on nuclear medicine practice around the world,^2^ in which the total number of therapies in different countries is included. However, this database, intended mainly for developing countries, is compiled on a voluntary basis. As a result, the data are by no means complete and for many European countries are below the number of therapies reported in this survey. Furthermore, information on the different kinds of MRTs is not available in the IAEA database. In 1999, the EANM Radionuclide Therapy Task Group reported on results from two questionnaires, distributed in 1993 to national advisors or members of the task group, with data acquired from 16 and 20 European countries [3]. The number of centers administering MRT in different countries was reported, identifying a total of 630 centers. In addition to that survey [3], a few national surveys have been identified [4, 5], as well as one reviewing nuclear medicine in the Middle East [6].

To address the shortage of information and put results of the current survey into perspective, the ITDF made an effort, after the survey was closed, to compile data of the number of centers that administer MRT in the countries from where responses were obtained (Table 5). The information sources identified were the EANM national delegates, national authorities or societies, published national data, and in some cases, personal communication or data retrieved from [3]. For the 25 countries where data could be obtained, a total of 925 centers were recorded. The 208 responders in the current survey were thus estimated to represent approximately 20% of the existing centers. For the future, a European database of centers performing MRT would need to be established in order to achieve complete overview of the situation. Similar initiatives have been undertaken in external beam radiotherapy as part of the ESTRO Health Economics in Radiation Oncology project [7].

**Table 5:** Number of centers that administer molecular radiotherapy in the countries from where responses were obtained. Numbers have been acquired from a variety of sources, as indicated, and are valid for 2015–2016 except where explicitly stated otherwise

Country	Number of centers	Source of information
Albania	No information	
Austria	7	Only number of inpatient facilities, personal communication
Belgium	143	Number of licensed sites. Registry of the Belgian Federal Agency for Nuclear Control
Bosnia and Herzegovina	3	EANM National Delegate
Bulgaria	11	EANM National Delegate
Croatia	6	EANM National Delegate
Cyprus	5	Ministry of Health (public hospitals) and personal communication (private hospitals)
Czech Republic	47	Institute of Health Information and Statistics of the Czech Republic (online)
Denmark	14	EANM National Delegate
France	60	Valid 1999, reference [3]
Germany	114	Valid 2012, reference [5]
Greece	33	Greek Atomic Energy Comission
Hungary	13	EANM National Delegate
Ireland	14	EANM National Delegate
Italy	106	Valid 2006, White paper of Italian Association of Nuclear Medicine (online)
Latvia	1	EANM National Delegate
Netherlands	66	Valid 2014, Dutch health research institute (online)
Norway	19	Norwegian Radiation Protection Authority
Portugal	32	Ministry of Health of Portugal
Serbia	7	EANM National Delegate
Slovakia	16	National Health Information Center (Slovakia)
Spain	90	National Safety Council of Spain
Sweden	24	Swedish Society of Radionuclide Therapy (online)
Switzerland	13	Only number of inpatient facilities, personal communication
Turkey	11	Valid 1999, reference [3]
United Kingdom	70	Estimated, personal communication

### Survey construction and dissemination

For this survey, there were evident challenges in reaching responders, resulting in an irregular response rate between countries (Fig. 1 and Table 5). Following the initial launch via the official route of dissemination, the response rate was rather modest, and we partly relied on national networks through which the survey could be forwarded. The construction of the survey lasted almost 1 year and built upon discussions in the ITDF, taking the different national perspectives into account, and the fact that different radionuclide therapies have different treatment aims, procedures for administration, level of patient hospitalization, profiles of treatment-related risks, and ease of implementation of dosimetry. During these discussions, a number of potential problems were identified, especially concerning the terminology that is not well established. For instance, the modality itself runs under various names such as “nuclear medicine therapy,” “radiopharmaceutical therapy,” and “(targeted) radionuclide therapy,” in addition to “molecular radiotherapy,” as used herein. Another ambiguous term is the word “treatment,” which for radionuclide therapies involving planned repeated administrations may refer to one administration or a series of administrations. Here, terms such as “fraction” or “treatment cycle” have sometimes been borrowed from other treatment modalities although these may be more or less suited within the context of particular therapies. The need to develop standardized terminology is thus evident. For the question concerning the involvement of a medical physicist, it was recognized that the word “involved” could be clarified further. However, also in the Council Directive (2013/59/Euratom), the word “involved” is being used without clarification, and seeing that one of the aims was to understand the compliance to that Directive, it was decided to maintain this wording. The prescription basis was particularly difficult to summarize in a few questions, in view of the quite different treatment protocols applied in different countries and within countries. To some extent, this can be seen as a result in its own, pointing to the diversity of treatment protocols that are being employed for patients in Europe.

### Results for treatments, the use of dosimetry, and the involvement of medical physicists

The most commonly performed therapies were clearly ^131^I-NaI for benign thyroid diseases and thyroid ablation of adults (Table 3, therapies A and B). These were also the therapies with largest geographical spread. As shown in Fig. 2, 54% of the responses stated that absorbed-dose planning was being undertaken in treatments of benign thyroid diseases in Always/Majority of treatments, while the same number, 54%, responded that a medical physicist was involved (Always/Majority). For ^131^I-NaI thyroid ablation of adults, 23% of the responses stated that absorbed-dose planning was undertaken and 21% that post-therapy dosimetry was performed in Always/Majority of treatments, while 55% responded that a medical physicist was involved (Always/Majority). Overall, this suggests that approximately 50% of the responding centers administer these treatments (A and B) without involvement of a medical physicist and without any dosimetry.

The most concerning results are perhaps those presented in Fig. 2a, showing an average frequency of the Minority/Never responses for involvement of a medical physicist of 32%. Without the involvement of a medical physicist, the likelihood that dosimetry can be undertaken is probably low. This may also explain the confusion that was occasionally encountered in the free-text replies, of the generic term dose (for instance in units of MBq), versus the SI-derived quantity absorbed dose (in unit Gy). The survey questions clearly distinguished between activity and absorbed dose and did not use the term “dose” without specification. Thus, the possible confusion between administered activity and absorbed dose was not likely caused by the wording of the survey questions. However, seeing that the general term “dose” is often used in nuclear medicine, misunderstandings may still exist.

Among the therapies not involving ^131^I-NaI, ^223^RaCl_2_ therapies for bone metastases accounted for 3.3% of the total patients treated (data not shown). Given that repeated administrations are foreseen in the therapy protocol [8], this corresponded to 10% of the total radionuclide treatments in our survey (Table 3, therapy P). Concerning the geographical spread, 13 of the countries from which responses were obtained gave this treatment. This is a comparably high number in view of the short history of this therapy. Although imaging and dosimetry of ^223^RaCl_2_ presents several challenges [9–11], 38% of centers responded that the absorbed dose was individually planned, 17% that post-therapy imaging was performed, and 4% that post-therapy dosimetry was performed, on an Always/Majority basis. In view of the practical difficulties in performing absorbed-dose planning for ^223^RaCl_2_ and that 98% of responses on the activity prescription stated different kinds of fixed-activity protocols, these figures appear unlikely and raise the question on the understanding of the concept of individual-based absorbed-dose determination. It is possible that responders did not distinguish between an individually calculated absorbed dose and the absorbed-dose estimation per unit of administered activity available in the package insert.

Other comparably frequent treatments were ^177^Lu or ^90^Y PRRT (Table 3, therapies H and I). PRRTs are given as repeated administrations, and while the percentages of the treated patients were 3% (H) and 1% (I) (data not shown), the percentages of the given number of treatments were 6% (H) and 5% (I). Concerning the geographical spread, 13 out of the 26 countries were administering either of these treatments. This is a relatively high proportion considering that neither of these therapies is yet approved for clinical use by the European Medicines Agency (EMA). For the responses Always/Majority (Fig. 2), 15% (H) and 33% (I) of centers responded that the absorbed dose was individually planned, 100% (H) and 89% (I) that post-therapy imaging was performed, and 44% (H) and 39% (I) that post-therapy dosimetry was performed. These results appear somewhat more consistent than for ^223^RaCl_2_, since the practical implementation of imaging and post-therapy dosimetry is more straightforward [12, 13], and since without EMA approval, these therapies are probably to a larger extent administered as part of clinical trials.

**Fig. 4:**
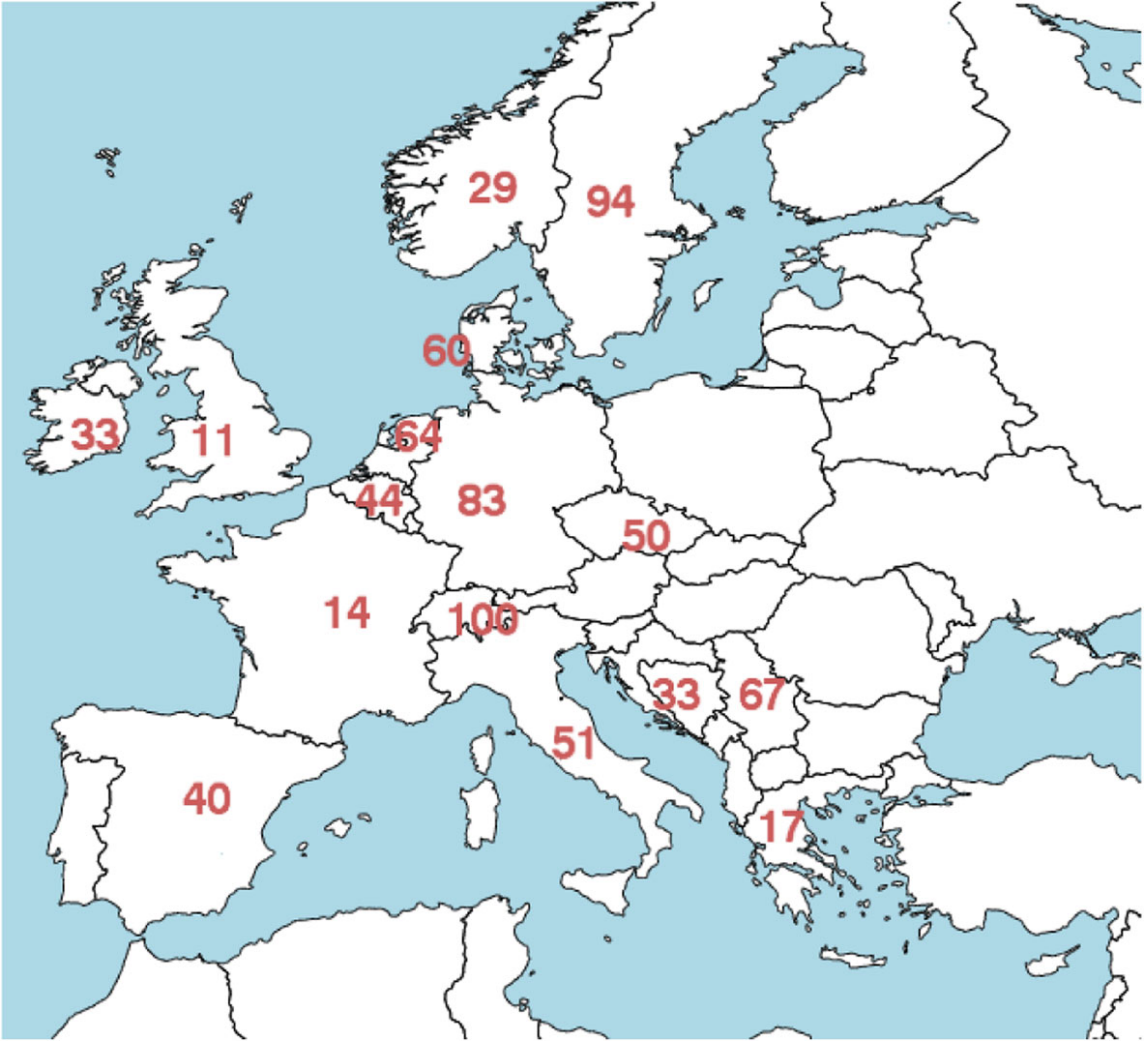
Percentage of responses from different countries who stated Always or Majority on the question “Is the absorbed dose individually planned for each patient?” for ^131^I-NaI treatment of benign thyroid diseases. Only countries represented by 3 or more responders are included

The most frequent application of any dosimetry, i.e., responses in the Always/Majority category either as part of treatment planning or on a post-therapy basis (Fig. 2), was reported for ^177^Lu-PSMA (100%), ^90^Y microspheres of glass (84%) and resin (82%), ^131^I-mIBG for neuroblastoma (59%), and ^131^I-NaI for benign thyroid diseases (54%). Possible explanations for these results are that ^177^Lu-PSMA is yet not approved for clinical use by the EMA and is thus being evaluated as part of clinical trials in a limited number of centers (2 of the 26 countries). For ^90^Y microspheres, the risks for radiation-induced liver disease are of clinical concern, and at the same time, patient-specific dosimetry can be accomplished using only one patient scan [14]. Dosimetry in ^131^I-mIBG for neuroblastoma is particularly motivated in view of the pediatric/young patient population. The implementation of dosimetry in ^131^I-NaI treatments for benign thyroid disease is compulsory in some countries, which may explain the geographical variability, as further highlighted in Fig. 4.

### Free-text comments from the responders

The free-text fields were available on the survey for collecting additional comments from the participants. For instance, some pointed at the lack of information on the tolerance levels of normal organs as an obstacle for clinical implementation of dosimetry, while others argued that prospective, randomized trials for examining the clinical value of dosimetry are needed before implementation. Others pointed at needs for resources and equipment, methodological guidance, and standardization of dosimetry methods. According to the questions on user satisfaction, 45% of the responders were satisfied with the implementation of dosimetry in their center. Interestingly, 12% of responders stated that a limiting factor was that there is no legislative requirement to perform dosimetry.

### Summary

In summary, our European survey showed a wide variation in MRT and dosimetry practices. Although MRT has a long history of being prescribed based on different fixed-activity schemas, with successful treatment results obtained in patient groups, this does not mean that the treatment is also optimal for an individual patient. For instance, it has been demonstrated that, for a given administered activity, the absorbed dose to target tissue may vary by more than two orders of magnitude for the same treatment [15]. Correlations between estimated absorbed doses and clinical outcomes have been reported for several types of MRTs [16–22], and some therapeutic prescriptions are already based on dosimetry [23–28]. It is likely that additional therapeutic scenarios would benefit from dosimetry studies. In fact, for some therapies, fixed activities might be too conservative, and patient-based dosimetry might indicate the need for activity escalation in order to achieve the desired dose to the target. On the other hand, in some other cases, dosimetry studies might constrain the activity prescriptions to keep the absorbed doses to non-target organs within safe limits or rule out patients who would not achieve enough absorbed doses to the target, helping to balance risks and benefits on a single patient basis. Furthermore, as pointed out already in 1992 [29], there may be economical incentives for implementation of dosimetry. These authors reported on results of dosimetry-based ^131^I-NaI thyroid cancer therapy for adults and found that the savings made on a lower rate of hospitalization well balanced the extra costs for dosimetry. Bearing in mind that we are in an era when personalized treatments are the focus of many kinds of radiotherapy, as well as pharmaceutical therapies, MRT has an extraordinary advantage in that the pharmaceutical agent and its distribution over time can be monitored and quantified.

## Conclusion

There is a wide variation in MRT practice across Europe, including the extent of medical-physicist involvement and the implementation of dosimetry-guided treatments. In many cases, dosimetry is feasible, and several therapies exist in which dosimetry is being performed on a fairly standardized basis in different centers and countries. However, in respect of the Council Directive (2013/59/Euratom), it appears that there is still a general need to increase the possibilities and benefits of a higher degree of implementation of dosimetry.

## Additional file

Additional file 1: IDTF electronic version of survey results submission and EANM Internal Dosimetry Task Force Survey. (ZIP 801 kb)

## Abbreviations

EANM: European association of nuclear medicine
IDTF: Internal dosimetry task force
MRT: Molecular radiotherapy

## Footnotes

1 See http://www.eanm.org/about/organs/national-societies/ for a complete list of national societies.

2
http://nucmedicine.iaea.org/
