# Supplementary material for: Variations in the practice of molecular radiotherapy and implementation of dosimetry: results from a European survey
Source: EJNMMI Phys. 2017 Dec 4;4:28. doi: 10.1186/s40658-017-0193-4 (PMC5712507; doi:10.1186/s40658-017-0193-4)
Supplement: Additional file 1: — IDTF electronic version of survey results submission and EANM Internal Dosimetry Task Force Survey. (ZIP 801 kb) [file 40658_2017_193_MOESM1_ESM.zip › EANM Internal Dosimetry Task Force Survey.pdf]

EANM Internal Dosimetry Task Force Survey

The EANM Internal Dosimetry Task Force (IDTF) is a cross-committee group formed to address aspects of the Euratom 2013/59 directive specifically concerned with dosimetry for radionuclide therapy. The aim of this survey is to determine the range and number of therapy procedures performed in Europe.

The survey consists of a single page for each therapy procedure, and includes questions identical or similar to those found in this example form goo.gl/NxRzhg. Each page should take about 3 minutes to complete.

IMPORTANT! The survey can be saved for completion at a later date. On return, it is necessary to fill in personal details once again and then select the right section to start from. The data previously entered will NOT be shown on the webpage (as this feature is not currently supported by Google form). In order to correctly identify your responses and aggregate all the entries it is important that you use EXACTLY the same personal details every time you enter the survey.

The survey is constructed as a series of pages, each relating to a particular therapy:

A. 131I NaI for benign thyroid diseases
B. 131I NaI for thyroid ablation of adults
C. 131I NaI for thyroid ablation of children and young adults
D. 131I NaI for thyroid cancer therapy for adults
E. 131I NaI for thyroid cancer therapy for children and young adults
F. 131I mIBG for neuroblastoma
G. 131I mIBG for adult neuroendocrine tumours
H. 177Lu Somatostatin Receptor Radionuclide Therapy
I. 90Y Somatostatin Receptor Radionuclide Therapy
J. 177Lu PSMA therapy of castration-resistant prostate cancer
K. 90Y resin microspheres for intra-arterial treatments in the liver
L. 90Y glass microspheres for intra-arterial treatments in the liver
M. Radiation synovectomy using 90Y, 186Re or 169Er
N. 153Sm for bone metastases
O. 89Sr for bone metastases
P. 223Ra for bone metastases
Q. 32P phosphate for myeloproliferative diseases
R. 90Y Zevalin for B-cell lymphoma
S. Therapy using alpha-emitting radionuclides other than 223Ra (option 1)
T. Therapy using alpha-emitting radionuclides other than 223Ra (option 2)
U. Therapy using alpha-emitting radionuclides other than 223Ra (option 3)
V. Therapy using other radiopharmaceutical (option 1)
W. Therapy using other radiopharmaceutical (option 2)
X. Therapy using other radiopharmaceutical (option 3)
Y. General questions

We do ask for your contact details although the survey results will be anonymised prior to dissemination.

The information from this survey will be used to promote European growth and collaboration as this area expands.

Thank you for your time and understanding

The EANM Dosimetry Task Force

*Required

Participant's data

1. **First Name** *

2. **Last Name** *

3. **Working place** *

4. **City** *

5. **Country** *

Mark only one oval.

- Albania
- Andorra
- Armenia
- Austria
- Azerbaijan
- Belarus
- Belgium
- Bosnia and Herzegovina
- Bulgaria
- Croatia
- Cyprus
- Czech Republic
- Denmark
- Estonia
- Finland
- France
- Georgia
- Germany
- Greece
- Hungary
- Iceland
- Ireland
- Italy
- Kazakhstan
- Kosovo
- Latvia
- Liechtenstein
- Lithuania
- Luxembourg
- Macedonia
- Malta
- Moldova
- Monaco
- Montenegro
- Netherlands
- Norway
- Poland

- Portugal
- Romania
- Russia
- San Marino
- Serbia
- Slovakia
- Slovenia
- Spain
- Sweden
- Switzerland
- Turkey
- Ukraine
- United Kingdom
- Vatican City (Holy See)

6. **E-mail** *

____

7. **I agree that our data can be presented in public, when data are anonymized but traceable to our country** *

Mark only one oval.

- YES
- NO

Welcome to the EANM IDTF Survey

8. **Where do you want to start?**

Select the section you want to start from.
Mark only one oval.

- Start form the beginning (new survey)
- Continue from Section: A. NaI for benign thyroid diseases *Skip to question 9.*
- Continue from Section: B. 131I NaI for thyroid ablation of adults *Skip to question 26.*
- Continue from Section: C. 131I NaI for thyroid ablation of children and young adults *Skip to question 41.*
- Continue from Section: D. 131I NaI for thyroid cancer therapy for adults *Skip to question 56.*
- Continue from Section: E. 131I NaI for thyroid cancer therapy for children and young adults *Skip to question 73.*
- Continue from Section: F. 131I mIBG for neuroblastoma *Skip to question 90.*
- Continue from Section: G. 131I mIBG for adult neuroendocrine tumors *Skip to question 105.*
- Continue from Section: H. 177Lu Somatostatin Receptor Radionuclide Therapy *Skip to question 121.*
- Continue from Section: I. 90Y Somatostatin Receptor Radionuclide Therapy *Skip to question 138.*
- Continue from Section: J. 177Lu PSMA therapy of castration-resistant prostate cancer *Skip to question 154.*
- Continue from Section: K. 90Y resin microspheres for intra-arterial treatments in the liver *Skip to question 169.*
- Continue from Section: L. 90Y glass microspheres for intra-arterial treatments in the liver *Skip to question 183.*
- Continue from Section: M. Radiation synovectomy using 90Y, 186Re or 169Er *Skip to question 196.*
- Continue from Section: N. 153Sm for bone metastases *Skip to question 222.*
- Continue from Section: O. 89Sr for bone metastases *Skip to question 238.*
- Continue from Section: P. 223Ra for bone metastases *Skip to question 254.*
- Continue from Section: Q. 32P phosphate for myeloproliferative diseases *Skip to question 270.*
- Continue from Section: R. 90Y Zevalin for B-cell lymphoma *Skip to question 286.*
- Continue from Section: S. Therapy using alpha-emitting radionuclides other than 223Ra (option 1) *Skip to question 298.*
- Continue from Section: T. Therapy using alpha-emitting radionuclides other than 223Ra (option 2) *Skip to question 313.*
- Continue from Section: U. Therapy using alpha-emitting radionuclides other than 223Ra (option 3) *Skip to question 328.*
- Continue from Section: V. Therapy using other radiopharmaceutical (option 1) *Skip to question 343.*
- Continue from Section: W. Therapy using other radiopharmaceutical (option 2) *Skip to question 358.*
- Continue from Section: X. Therapy using other radiopharmaceutical (option 3)

Skip to question 373.

- Continue from Section: Y. General questions *Skip to question 388.*

A. 131I NaI for benign thyroid diseases

9. **Number of patients treated in the year 2015**

____

10. **Number of treatments performed in 2015**

____

11. **Which medical specialty owns the license to administer treatment?**
Tick all that apply.

- [ ] Nuclear Medicine
- [ ] Medical Oncology
- [ ] Radiation Oncology
- [ ] Endocrinology
- [ ] Interventional Radiology
- [ ] Other: ________________

12. **Is a medical physicist involved in each treatment?**
Mark only one oval.

- Always
- Majority
- Minority
- Never

13. **Is the absorbed dose individually planned for each patient?**
Mark only one oval.

- Always
- Majority
- Minority
- Never

14. **Is post-therapy imaging performed?**
Mark only one oval.

- Always
- Majority
- Minority
- Never

15. **Is post-therapy dosimetry performed?**
Mark only one oval.

- Always
- Majority
- Minority
- Never

16. **What basis of prescription do you typically use (specify in below box)**
Tick all that apply.

- Fixed activity
- Fixed activity based on diagnosis, stage, or other clinical factors
- Fixed activity per body surface area or patient weight
- Individually calculated activity to give a prescribed absorbed dose to whole body
- Individually calculated activity to give a prescribed absorbed dose to organ at risk
- Individually calculated activity to give a prescribed absorbed dose to target tissue
- Other prescription (specify below)

17. **Please specify the prescribed AMOUNT for toxic or nontoxic multinodular goitre**

18. **Please specify the UNIT of the prescription**
Mark only one oval.

- MBq
- MBq / kg
- MBq / m2
- Gy

19. **Please specify the prescribed AMOUNT for autonomous nodules**

20. **Please specify the UNIT of the prescription**
Mark only one oval.

- MBq
- MBq / kg
- MBq / m2
- Gy

21. **Please specify the prescribed AMOUNT for Graves' disease**

22. **Please specify the UNIT of the prescription**
Mark only one oval.

- MBq
- MBq / kg
- MBq / m2
- Gy

23. **Please add any additional information regarding the prescription**

24. **Is therapy repeated when the first treatment is insufficient?**
Mark only one oval.

- Always
- Majority
- Minority
- Never

25. **What would you like to do next ?**
If you choose to save your results and quit for now (1st alternative) you will be taken to the submission page. When you are ready to continue with the survey please go back to the homepage, re-insert your contact details and choose the section you want to continue from. If you want to go ahead with the survey now, you can either choose which section to go to, or continue to the next page.
Mark only one oval.

- Quit for now, take me to the submission page *Skip to "Thank you."*
- Let me choose which section to go to *Skip to question 8.*
- Take me to the next page

B. 131I NaI for thyroid ablation of adults

26. **Number of patients treated in the year 2015**

27. **Number of treatments performed in 2015**

28. **Which medical specialty owns the license to administer treatment?**
Tick all that apply.

- [ ] Nuclear Medicine
- [ ] Medical Oncology
- [ ] Radiation Oncology
- [ ] Endocrinology
- [ ] Interventional Radiology
- [ ] Other: ____________________

29. **Is a medical physicist involved in each treatment?**
Mark only one oval.

- ( ) Always
- ( ) Majority
- ( ) Minority
- ( ) Never

30. **Is the absorbed dose individually planned for each patient?**
Mark only one oval.

- ( ) Always
- ( ) Majority
- ( ) Minority
- ( ) Never

31. **Is post-therapy imaging performed?**
Mark only one oval.

- ( ) Always
- ( ) Majority
- ( ) Minority
- ( ) Never

32. **Is post-therapy dosimetry performed?**
Mark only one oval.

- ( ) Always
- ( ) Majority
- ( ) Minority
- ( ) Never

33. **What basis of prescription do you typically use (specify in below box)**
Tick all that apply.

- [ ] Fixed activity
- [ ] Fixed activity based on diagnosis, stage, or other clinical factors
- [ ] Fixed activity per body surface area or patient weight
- [ ] Individually calculated activity to give a prescribed absorbed dose to whole body
- [ ] Individually calculated activity to give a prescribed absorbed dose to organ at risk
- [ ] Individually calculated activity to give a prescribed absorbed dose to target tissue
- [ ] Other prescription (specify below)

34. **Please specify the Lowest prescribed AMOUNT**

____

35. **Please specify the UNIT of the prescription**
Mark only one oval.

- ( ) MBq
- ( ) MBq / kg
- ( ) MBq / m2
- ( ) Gy

36. **Please specify the Highest prescribed AMOUNT**

____

37. **Please specify the UNIT of the prescription**
Mark only one oval.

- ( ) MBq
- ( ) MBq / kg
- ( ) MBq / m2
- ( ) Gy

38. **Please add any additional information regarding the prescription**

____

____

____

____

____

39. **Is rhTSH stimulation applied before therapy?**
Mark only one oval.

- Always
- Majority
- Minority
- Never

40. **What would you like to do next ?**
If you choose to save your results and quit for now (1st alternative) you will be taken to the submission page. When you are ready to continue with the survey please go back to the homepage, re-insert your contact details and choose the section you want to continue from. If you want to go ahead with the survey now, you can either choose which section to go to, or continue to the next page.
Mark only one oval.

- Quit for now, take me to the submission page *Skip to "Thank you."*
- Let me choose which section to go to *Skip to question 8.*
- Take me to the next page

C. 131I NaI for thyroid ablation of children and young adults (<18 years old)

41. **Number of patients treated in the year 2015**

42. **Number of treatments performed in 2015**

43. **Which medical specialty owns the license to administer treatment?**
Tick all that apply.

- Nuclear Medicine
- Medical Oncology
- Radiation Oncology
- Endocrinology
- Interventional Radiology
- Other:

44. **Is a medical physicist involved in each treatment?**
Mark only one oval.

- Always
- Majority
- Minority
- Never

45. **Is the absorbed dose individually planned for each patient?**
Mark only one oval.

- Always
- Majority
- Minority
- Never

46. **Is post-therapy imaging performed?**
Mark only one oval.

- Always
- Majority
- Minority
- Never

47. **Is post-therapy dosimetry performed?**
Mark only one oval.

- Always
- Majority
- Minority
- Never

48. **What basis of prescription do you typically use (specify in below box)**
Tick all that apply.

- [ ] Fixed activity
- [ ] Fixed activity based on diagnosis, stage, or other clinical factors
- [ ] Fixed activity per body surface area or patient weight
- [ ] Individually calculated activity to give a prescribed absorbed dose to whole body
- [ ] Individually calculated activity to give a prescribed absorbed dose to organ at risk
- [ ] Individually calculated activity to give a prescribed absorbed dose to target tissue
- [ ] Other prescription (specify below)

49. **Please specify the Lowest prescribed AMOUNT**

50. **Please specify the UNIT of the prescription**
Mark only one oval.

- MBq
- MBq / kg
- MBq / m2
- Gy

51. **Please specify the Highest prescribed AMOUNT**

52. **Please specify the UNIT of the prescription**
Mark only one oval.
- MBq
- MBq / kg
- MBq / m2
- Gy

53. **Please add any additional information regarding the prescription**

54. **Is rhTSH stimulation applied before therapy?**
Mark only one oval.
- Always
- Majority
- Minority
- Never

55. **What would you like to do next ?**
If you choose to save your results and quit for now (1st alternative) you will be taken to the submission page. When you are ready to continue with the survey please go back to the homepage, re-insert your contact details and choose the section you want to continue from. If you want to go ahead with the survey now, you can either choose which section to go to, or continue to the next page.
Mark only one oval.
- Quit for now, take me to the submission page *Skip to "Thank you."*
- Let me choose which section to go to *Skip to question 8.*
- Take me to the next page

D. 131I NaI for thyroid cancer (post-ablative or metastatic) therapy for adults

56. **Number of patients treated in the year 2015**

57. **Number of treatments performed in 2015**

____

58. **Which medical specialty owns the license to administer treatment?**
Tick all that apply.

- [ ] Nuclear Medicine
- [ ] Medical Oncology
- [ ] Radiation Oncology
- [ ] Endocrinology
- [ ] Interventional Radiology
- [ ] Other: ____________________

59. **Is a medical physicist involved in each treatment?**
Mark only one oval.

- ( ) Always
- ( ) Majority
- ( ) Minority
- ( ) Never

60. **Is the absorbed dose individually planned for each patient?**
Mark only one oval.

- ( ) Always
- ( ) Majority
- ( ) Minority
- ( ) Never

61. **Is post-therapy imaging performed?**
Mark only one oval.

- ( ) Always
- ( ) Majority
- ( ) Minority
- ( ) Never

62. **Is post-therapy dosimetry performed?**
Mark only one oval.

- ( ) Always
- ( ) Majority
- ( ) Minority
- ( ) Never

63. **What basis of prescription do you typically use (specify in below box)**
Tick all that apply.

- [ ] Fixed activity
- [ ] Fixed activity based on diagnosis, stage, or other clinical factors
- [ ] Fixed activity per body surface area or patient weight
- [ ] Individually calculated activity to give a prescribed absorbed dose to whole body
- [ ] Individually calculated activity to give a prescribed absorbed dose to organ at risk
- [ ] Individually calculated activity to give a prescribed absorbed dose to target tissue
- [ ] Other prescription (specify below)

64. **Please specify the Lowest prescribed AMOUNT**

65. **Please specify the UNIT of the prescription**
Mark only one oval.

- ( ) MBq
- ( ) MBq / kg
- ( ) MBq / m2
- ( ) Gy

66. **Please specify the Highest prescribed AMOUNT**

67. **Please specify the UNIT of the prescription**
Mark only one oval.

- ( ) MBq
- ( ) MBq / kg
- ( ) MBq / m2
- ( ) Gy

68. **Please add any additional information regarding the prescription**

69. **If repeated therapies are required, on what basis would repetition be discontinued?**
Tick all that apply.

- [ ] Evidence of undifferentiated disease (i.e. absence of iodine uptake despite radiological or biochemical evidence of disease)
- [ ] Absence of biochemical disease (i.e. both Tg and anti-Tg AB negativity)
- [ ] Achievement of stable biochemical markers within a certain limit, or within acceptable limits (please specify in box below) *
- [ ] Cumulative administered activity (or total body dose) beyond a certain limit, or beyond an acceptable risk-benefit ratio (please specify in box below)*
- [ ] Need for surgical or radiotherapy de-bulking
- [ ] Other criteria (specify below)

70. **Please specify other criteria**

71. **In case alternatives (*) are selected above, please specify what acceptance limits are applied for Tg levels/antiTg AB levels, and/or for cumulative activity (or dose)/risk-benefit ratio:**

72. **What would you like to do next ?**
If you choose to save your results and quit for now (1st alternative) you will be taken to the submission page. When you are ready to continue with the survey please go back to the homepage, re-insert your contact details and choose the section you want to continue from. If you want to go ahead with the survey now, you can either choose which section to go to, or continue to the next page.
Mark only one oval.

- ( ) Quit for now, take me to the submission page *Skip to "Thank you."*
- ( ) Let me choose which section to go to *Skip to question 8.*
- ( ) Take me to the next page

E. 131I NaI for thyroid cancer (post-ablative or metastatic) therapy for children and young adults (<18 years old)

73. **Number of patients treated in the year 2015**

74. **Number of treatments performed in 2015**

75. **Which medical specialty owns the license to administer treatment?**
Tick all that apply.

- [ ] Nuclear Medicine
- [ ] Medical Oncology
- [ ] Radiation Oncology
- [ ] Endocrinology
- [ ] Interventional Radiology
- [ ] Other:

76. **Is a medical physicist involved in each treatment?**
Mark only one oval.

- Always
- Majority
- Minority
- Never

77. **Is the absorbed dose individually planned for each patient?**
Mark only one oval.

- Always
- Majority
- Minority
- Never

78. **Is post-therapy imaging performed?**
Mark only one oval.

- Always
- Majority
- Minority
- Never

79. **Is post-therapy dosimetry performed?**
Mark only one oval.

- Always
- Majority
- Minority
- Never

80. **What basis of prescription do you typically use (specify in below box)**
Tick all that apply.

- [ ] Fixed activity
- [ ] Fixed activity based on diagnosis, stage, or other clinical factors
- [ ] Fixed activity per body surface area or patient weight
- [ ] Individually calculated activity to give a prescribed absorbed dose to whole body
- [ ] Individually calculated activity to give a prescribed absorbed dose to organ at risk
- [ ] Individually calculated activity to give a prescribed absorbed dose to target tissue
- [ ] Other prescription (specify below)

81. **Please specify the Lowest prescribed AMOUNT**

____

82. **Please specify the UNIT of the prescription**
Mark only one oval.

- MBq
- MBq / kg
- MBq / m2
- Gy

83. **Please specify the Highest prescribed AMOUNT**

____

84. **Please specify the UNIT of the prescription**
Mark only one oval.

- MBq
- MBq / kg
- MBq / m2
- Gy

85. **Please add any additional information regarding the prescription**

86. **If repeated therapies are required, on what basis would repetition be discontinued?**
Tick all that apply.

- [ ] Evidence of undifferentiated disease (i.e. absence of iodine uptake despite radiological or biochemical evidence of disease)
- [ ] Absence of biochemical disease (i.e. both Tg and anti-Tg AB negativity)
- [ ] Achievement of stable biochemical markers within a certain limit, or within acceptable limits (please specify in box below)*
- [ ] Cumulative administered activity (or total body dose) beyond a certain limit, or beyond an acceptable risk-benefit ratio (please specify in box below)*
- [ ] Need for surgical or radiotherapy de-bulking
- [ ] Other: ____________

87. **Please specify other criteria**

88. **In case alternatives (*) are selected above, please specify what acceptance limits are applied for Tg levels/antiTg AB levels, and/or for cumulative activity (or dose)/risk-benefit ratio**

89. **What would you like to do next ?**

If you choose to save your results and quit for now (1st alternative) you will be taken to the submission page. When you are ready to continue with the survey please go back to the homepage, re-insert your contact details and choose the section you want to continue from. If you want to go ahead with the survey now, you can either choose which section to go to, or continue to the next page.

Mark only one oval.

- Quit for now, take me to the submission page *Skip to "Thank you."*
- Let me choose which section to go to *Skip to question 8.*
- Take me to the next page

F. 131I mIBG for neuroblastoma

90. **Number of patients treated in the year 2015**

91. **Number of treatments performed in 2015**

92. **Which medical specialty owns the license to administer treatment?**

Tick all that apply.

- Nuclear Medicine
- Medical Oncology
- Radiation Oncology
- Endocrinology
- Interventional Radiology
- Other:

93. **Is a medical physicist involved in each treatment?**

Mark only one oval.

- Always
- Majority
- Minority
- Never

94. **Is the absorbed dose individually planned for each patient?**

Mark only one oval.

- Always
- Majority
- Minority
- Never

95. **Is post-therapy imaging performed?**
Mark only one oval.

- Always
- Majority
- Minority
- Never

96. **Is post-therapy dosimetry performed?**
Mark only one oval.

- Always
- Majority
- Minority
- Never

97. **What basis of prescription do you typically use (specify in below box)**
Tick all that apply.

- [ ] Fixed activity
- [ ] Fixed activity based on diagnosis, stage, or other clinical factors
- [ ] Fixed activity per body surface area or patient weight
- [ ] Individually calculated activity to give a prescribed absorbed dose to whole body
- [ ] Individually calculated activity to give a prescribed absorbed dose to organ at risk
- [ ] Individually calculated activity to give a prescribed absorbed dose to target tissue
- [ ] Other prescription (specify below)

98. **Please specify the prescribed AMOUNT**

____

99. **Please specify the UNIT of the prescription**
Mark only one oval.

- MBq
- MBq / kg
- MBq / m2
- Gy

100. **Please add any additional information regarding the prescription**

____
____
____
____
____

101. **Is therapy prescribed as repeated administrations?**
Mark only one oval.

- Always
- Majority
- Minority
- Never

102. **If repeated administrations are given, how many?**
Mark only one oval.

- 2
- 3
- 4
- 5
- 6
- 7
- 8
- 9
- 10

103. **If repeated administrations are given, with which time interval between them (weeks)?**
Mark only one oval.

- 1
- 2
- 3
- 4
- 5
- 6
- 7
- 8
- 9
- 10
- 11
- 12
- >12

104. **What would you like to do next ?**

If you choose to save your results and quit for now (1st alternative) you will be taken to the submission page. When you are ready to continue with the survey please go back to the homepage, re-insert your contact details and choose the section you want to continue from. If you want to go ahead with the survey now, you can either choose which section to go to, or continue to the next page.

Mark only one oval.

- Quit for now, take me to the submission page *Skip to "Thank you."*
- Let me choose which section to go to *Skip to question 8.*
- Take me to the next page

G. 131I mIBG for adult neuroendocrine tumours

105. **Number of patients treated in the year 2015**

106. **Number of treatments performed in 2015**

107. **Which medical specialty owns the license to administer treatment?**

Tick all that apply.

- Nuclear Medicine
- Medical Oncology
- Radiation Oncology
- Endocrinology
- Interventional Radiology
- Other:

108. **Indications (tumor type) for which treatment is given**

Tick all that apply.

- Phaeochromocytoma
- Paraganglioma
- Carcinoid tumour
- Medullary thyroid carcinoma
- Other:

109. **Is a medical physicist involved in each treatment?**

Mark only one oval.

- Always
- Majority
- Minority
- Never

110. **Is the absorbed dose individually planned for each patient?**
Mark only one oval.

- Always
- Majority
- Minority
- Never

111. **Is post-therapy imaging performed?**
Mark only one oval.

- Always
- Majority
- Minority
- Never

112. **Is post-therapy dosimetry performed?**
Mark only one oval.

- Always
- Majority
- Minority
- Never

113. **What basis of prescription do you typically use (specify in below box)**
Tick all that apply.

- [ ] Fixed activity
- [ ] Fixed activity based on diagnosis, stage, or other clinical factors
- [ ] Fixed activity per body surface area or patient weight
- [ ] Individually calculated activity to give a prescribed absorbed dose to whole body
- [ ] Individually calculated activity to give a prescribed absorbed dose to organ at risk
- [ ] Individually calculated activity to give a prescribed absorbed dose to target tissue
- [ ] Other prescription (specify below)

114. **Please specify the prescribed AMOUNT**

____

115. **Please specify the UNIT of the prescription**
Mark only one oval.

- MBq
- MBq / kg
- MBq / m2
- Gy

116. **Please add any additional information regarding the prescription**

117. **Is therapy prescribed as repeated administrations?**
Mark only one oval.

- Always
- Majority
- Minority
- Never

118. **If repeated administrations are given, how many?**
Mark only one oval.

- 2
- 3
- 4
- 5
- 6
- 7
- 8
- 9
- 10

119. **If repeated administrations are given, with which time interval between them (weeks)?**

Mark only one oval.

- 1
- 2
- 3
- 4
- 5
- 6
- 7
- 8
- 9
- 10
- 11
- 12
- >12

120. **What would you like to do next ?**

If you choose to save your results and quit for now (1st alternative) you will be taken to the submission page. When you are ready to continue with the survey please go back to the homepage, re-insert your contact details and choose the section you want to continue from. If you want to go ahead with the survey now, you can either choose which section to go to, or continue to the next page.

Mark only one oval.

- Quit for now, take me to the submission page *Skip to "Thank you."*
- Let me choose which section to go to *Skip to question 8.*
- Take me to the next page

H. 177Lu Somatostatin Receptor Radionuclide Therapy

121. **Number of patients treated in the year 2015**

____

122. **Number of treatments performed in 2015**

____

123. **Which medical specialty owns the license to administer treatment?**
Tick all that apply.

- [ ] Nuclear Medicine
- [ ] Medical Oncology
- [ ] Radiation Oncology
- [ ] Endocrinology
- [ ] Interventional Radiology
- [ ] Other: ______________________

124. **Indications (tumour type) for which treatment is given**
Tick all that apply.

- [ ] GEP-NETs
- [ ] Lung Carcinoids
- [ ] Small-cell lung cancer
- [ ] Medullary thyroid carcinoma
- [ ] Neuroblastoma
- [ ] Meningioma
- [ ] Merkel cell carcinoma
- [ ] Other: ______________________

125. **Is a medical physicist involved in each treatment?**
Mark only one oval.

- ( ) Always
- ( ) Majority
- ( ) Minority
- ( ) Never

126. **Is the absorbed dose individually planned for each patient?**
Mark only one oval.

- ( ) Always
- ( ) Majority
- ( ) Minority
- ( ) Never

127. **Is post-therapy imaging performed?**
Mark only one oval.

- ( ) Always
- ( ) Majority
- ( ) Minority
- ( ) Never

128. **Is post-therapy dosimetry performed?**
Mark only one oval.

- Always
- Majority
- Minority
- Never

129. **What basis of prescription do you typically use (specify in below box)**
Tick all that apply.

- Fixed activity
- Fixed activity based on diagnosis, stage, or other clinical factors
- Fixed activity per body surface area or patient weight
- Individually calculated activity to give a prescribed absorbed dose to whole body
- Individually calculated activity to give a prescribed absorbed dose to organ at risk
- Individually calculated activity to give a prescribed absorbed dose to target tissue
- Other prescription (specify below)

130. **Please specify the prescribed AMOUNT**

131. **Please specify the UNIT of the prescription**
Mark only one oval.

- MBq
- MBq / kg
- MBq / m2
- Gy

132. **Please add any additional information regarding the prescription**

133. **Is therapy prescribed as repeated administrations?**
Mark only one oval.

- Always
- Majority
- Minority
- Never

134. **If repeated administrations are given, how many?**

Mark only one oval.

- 2
- 3
- 4
- 5
- 6
- 7
- 8
- 9
- 10
- 11
- 12
- > 12
- tailored to the patient depending on dosimetry

135. **If repeated administrations are given, with which time interval between them (weeks)?**

Mark only one oval.

- 2
- 3
- 4
- 5
- 6
- 7
- 8
- 9
- 10
- 11
- 12
- 13
- 14
- 15
- 16
- 17
- 18
- 19
- 20
- > 20

136. **Are 177Lu and 90Y Somatostatin Receptor Radionuclide Therapies used in tandem?**

Mark only one oval.

- Yes
- No

137. **What would you like to do next ?**

If you choose to save your results and quit for now (1st alternative) you will be taken to the submission page. When you are ready to continue with the survey please go back to the homepage, re-insert your contact details and choose the section you want to continue from. If you want to go ahead with the survey now, you can either choose which section to go to, or continue to the next page.

Mark only one oval.

- Quit for now, take me to the submission page *Skip to "Thank you."*
- Let me choose which section to go to *Skip to question 8.*
- Take me to the next page

I. 90Y Somatostatin Receptor Radionuclide Therapy

138. **Number of patients treated in the year 2015**

____

139. **Number of treatments performed in 2015**

____

140. **Which medical specialty owns the license to administer treatment?**

Tick all that apply.

- [ ] Nuclear Medicine
- [ ] Medical Oncology
- [ ] Radiation Oncology
- [ ] Endocrinology
- [ ] Interventional Radiology
- [ ] Other: ____________________

141. **Indications (tumour type) for which treatment is given**
Tick all that apply.

- [ ] GEP-NETs
- [ ] Lung Carcinoids
- [ ] Small-cell lung cancer
- [ ] Medullary thyroid carcinoma
- [ ] Neuroblastoma
- [ ] Meningioma
- [ ] Merkel cell carcinoma
- [ ] Other: ____________________

142. **Is a medical physicist involved in each treatment?**
Mark only one oval.

- ( ) Always
- ( ) Majority
- ( ) Minority
- ( ) Never

143. **Is the absorbed dose individually planned for each patient?**
Mark only one oval.

- ( ) Always
- ( ) Majority
- ( ) Minority
- ( ) Never

144. **Is post-therapy imaging performed?**
Mark only one oval.

- ( ) Always
- ( ) Majority
- ( ) Minority
- ( ) Never

145. **Is post-therapy dosimetry performed?**
Mark only one oval.

- ( ) Always
- ( ) Majority
- ( ) Minority
- ( ) Never

146. **What basis of prescription do you typically use (specify in below box)**
Tick all that apply.

- [ ] Fixed activity
- [ ] Fixed activity based on diagnosis, stage, or other clinical factors
- [ ] Fixed activity per body surface area or patient weight
- [ ] Individually calculated activity to give a prescribed absorbed dose to whole body
- [ ] Individually calculated activity to give a prescribed absorbed dose to organ at risk
- [ ] Individually calculated activity to give a prescribed absorbed dose to target tissue
- [ ] Other prescription (specify below)

147. **Please specify the prescribed AMOUNT**

148. **Please specify the UNIT of the prescription**
Mark only one oval.

- MBq
- MBq / kg
- MBq / m2
- Gy

149. **Please add any additional information regarding the prescription**

150. **Is therapy prescribed as repeated administrations?**
Mark only one oval.

- Always
- Majority
- Minority
- Never

151. **If repeated administrations are given, how many?**
Mark only one oval.

- 2
- 3
- 4
- 5
- 6
- 7
- 8
- 9
- 10
- 11
- 12
- > 12
- tailored to the patient depending on dosimetry

152. **If repeated administrations are given, with which time interval between them (weeks)?**
Mark only one oval.

- 2
- 3
- 4
- 5
- 6
- 7
- 8
- 9
- 10
- 11
- 12
- 13
- 14
- 15
- 16
- 17
- 18
- 19
- 20
- > 20

153. **What would you like to do next ?**

If you choose to save your results and quit for now (1st alternative) you will be taken to the submission page. When you are ready to continue with the survey please go back to the homepage, re-insert your contact details and choose the section you want to continue from. If you want to go ahead with the survey now, you can either choose which section to go to, or continue to the next page.

Mark only one oval.

- Quit for now, take me to the submission page *Skip to "Thank you."*
- Let me choose which section to go to *Skip to question 8.*
- Take me to the next page

J. 177Lu prostate-specific membrane antigen (PSMA) therapy of castration-resistant prostate cancer

154. **Number of patients treated in the year 2015**

155. **Number of treatments performed in 2015**

156. **Which medical specialty owns the license to administer treatment?**

Tick all that apply.

- Nuclear Medicine
- Medical Oncology
- Radiation Oncology
- Endocrinology
- Interventional Radiology
- Other:

157. **Is a medical physicist involved in each treatment?**

Mark only one oval.

- Always
- Majority
- Minority
- Never

158. **Is the absorbed dose individually planned for each patient?**

Mark only one oval.

- Always
- Majority
- Minority
- Never

159. **Is post-therapy imaging performed?**
Mark only one oval.

- Always
- Majority
- Minority
- Never

160. **Is post-therapy dosimetry performed?**
Mark only one oval.

- Always
- Majority
- Minority
- Never

161. **What basis of prescription do you typically use (specify in below box)**
Tick all that apply.

- [ ] Fixed activity
- [ ] Fixed activity based on diagnosis, stage, or other clinical factors
- [ ] Fixed activity per body surface area or patient weight
- [ ] Individually calculated activity to give a prescribed absorbed dose to whole body
- [ ] Individually calculated activity to give a prescribed absorbed dose to organ at risk
- [ ] Individually calculated activity to give a prescribed absorbed dose to target tissue
- [ ] Other prescription (specify below)

162. **Please specify the prescribed AMOUNT**

163. **Please specify the UNIT of the prescription**
Mark only one oval.

- MBq
- MBq / kg
- MBq / m2
- Gy

164. **Please add any additional information regarding the prescription**

165. **Is therapy prescribed as repeated administrations?**
Mark only one oval.

- Always
- Majority
- Minority
- Never

166. **If repeated administrations are given, how many?**
Mark only one oval.

- 2
- 3
- 4
- 5
- 6
- 7
- 8
- 9
- 10
- 11
- 12
- 13
- 14
- 15
- > 15
- Tailored to the patient depending on dosimetry

167. **If repeated administrations are given, with which time interval between them (weeks)?**

Mark only one oval.

- 1
- 2
- 3
- 4
- 5
- 6
- 7
- 8
- 9
- 10
- 11
- 12
- 13
- 14
- 15
- 16
- 17
- 18
- 19
- 20
- > 20

168. **What would you like to do next ?**

If you choose to save your results and quit for now (1st alternative) you will be taken to the submission page. When you are ready to continue with the survey please go back to the homepage, re-insert your contact details and choose the section you want to continue from. If you want to go ahead with the survey now, you can either choose which section to go to, or continue to the next page.

Mark only one oval.

- Quit for now, take me to the submission page *Skip to "Thank you."*
- Let me choose which section to go to *Skip to question 8.*
- Take me to the next page

K. 90Y resin microspheres for intra-arterial treatments in the liver

169. **Number of patients treated in the year 2015**

170. **Number of treatments performed in 2015**

____

171. **Which medical specialty owns the license to administer treatment?**
Tick all that apply.

- [ ] Nuclear Medicine
- [ ] Medical Oncology
- [ ] Radiation Oncology
- [ ] Endocrinology
- [ ] Interventional Radiology
- [ ] Other: ________________________

172. **Indications (tumour type) for which treatment is given**
Tick all that apply.

- [ ] Hepato-cellular carcinoma
- [ ] Colorectal liver metastases
- [ ] Neuroendocrine liver metastases
- [ ] Colangiocarcinoma
- [ ] Breast cancer metastases
- [ ] Other: ________________________

173. **Is a medical physicist involved in each treatment?**
Mark only one oval.

- ( ) Always
- ( ) Majority
- ( ) Minority
- ( ) Never

174. **Is the absorbed dose individually planned for each patient?**
Mark only one oval.

- ( ) Always
- ( ) Majority
- ( ) Minority
- ( ) Never

175. **Is post-therapy imaging performed?**
Mark only one oval.

- Always
- Majority
- Minority
- Never

176. **Is post-therapy dosimetry performed?**
Mark only one oval.

- Always
- Majority
- Minority
- Never

177. **What basis of prescription do you typically use (specify in below box)**
Tick all that apply.

- [ ] Fixed activity
- [ ] Fixed activity based on diagnosis, stage, or other clinical factors
- [ ] Body-Surface-Area (BSA) method with reductions
- [ ] Individually calculated activity based on prescribed absorbed dose to the target volume (lobe or segment) to be treated
- [ ] Individually calculated activity based on 99mTc MAA SPECT to give a prescribed absorbed dose to non-tumoral liver tissue
- [ ] Individually calculated activity based on 99mTc MAA SPECT to give a prescribed absorbed dose to tumour
- [ ] Other: ____________________

178. **Please add any additional information regarding the prescription**

____
____
____
____
____

179. **Is therapy repeated in the same region?**
Mark only one oval.

- Always
- Majority
- Minority
- Never

180. **If repeated therapies are performed, how many times at maximum?**
Mark only one oval.

- 1
- 2
- 3
- 4
- 5

181. **If repeated therapies are performed, with which minimum time interval between treatments (weeks)?**

Mark only one oval.

- 1
- 2
- 3
- 4
- 5
- 6
- 7
- 8
- 9
- 10
- 11
- 12
- 13
- 14
- 15
- 16
- 17
- 18
- 19
- 20
- 21
- 22
- 23
- 24
- 25
- 26
- 27
- 28
- 29
- 30
- >30

182. **What would you like to do next ?**
If you choose to save your results and quit for now (1st alternative) you will be taken to the submission page. When you are ready to continue with the survey please go back to the homepage, re-insert your contact details and choose the section you want to continue from. If you want to go ahead with the survey now, you can either choose which section to go to, or continue to the next page.
Mark only one oval.

- ( ) Quit for now, take me to the submission page *Skip to "Thank you."*
- ( ) Let me choose which section to go to *Skip to question 8.*
- ( ) Take me to the next page

L. 90Y glass microspheres for intra-arterial treatments in the liver

183. **Number of patients treated in the year 2015**

____

184. **Number of treatments performed in 2015**

____

185. **Which medical specialty owns the license to administer treatment?**
Tick all that apply.

- [ ] Nuclear Medicine
- [ ] Medical Oncology
- [ ] Radiation Oncology
- [ ] Endocrinology
- [ ] Interventional Radiology
- [ ] Other: ____________________

186. **Indications (tumour type) for which treatment is given**
Tick all that apply.

- [ ] Hepato-cellular carcinoma
- [ ] Colorectal liver metastases
- [ ] Neuroendocrine liver metastases
- [ ] Colangiocarcinoma
- [ ] Breast cancer metastases
- [ ] Other: ____________________

187. **Is a medical physicist involved in each treatment?**
Mark only one oval.

- Always
- Majority
- Minority
- Never

188. **Is the absorbed dose individually planned for each patient?**
Mark only one oval.

- Always
- Majority
- Minority
- Never

189. **Is post-therapy imaging performed?**
Mark only one oval.

- Always
- Majority
- Minority
- Never

190. **Is post-therapy dosimetry performed?**
Mark only one oval.

- Always
- Majority
- Minority
- Never

191. **What basis of prescription do you typically use (specify in below box)**
Tick all that apply.

- Fixed activity
- Fixed activity based on diagnosis, stage, or other clinical factors
- Body-Surface-Area (BSA) method with reductions
- Individually calculated activity based on prescribed absorbed dose to the target volume (lobe or segment) to be treated
- Individually calculated activity based on 99mTc MAA SPECT to give a prescribed absorbed dose to non-tumoral liver tissue
- Individually calculated activity based on 99mTc MAA SPECT to give a prescribed absorbed dose to tumour
- Other: ____________________

192. **Is therapy repeated in the same region?**

Mark only one oval.

- Always
- Majority
- Minority
- Never

193. **If repeated therapies are performed, how many times at maximum?**

Mark only one oval.

- 1
- 2
- 3
- 4
- 5

194. **If repeated therapies are performed, with which minimum time interval between treatments (weeks)?**

Mark only one oval.

- 1
- 2
- 3
- 4
- 5
- 6
- 7
- 8
- 9
- 10
- 11
- 12
- 13
- 14
- 15
- 16
- 17
- 18
- 19
- 20
- 21
- 22
- 23
- 24
- 25
- 26
- 27
- 28
- 29
- 30
- >30

195. **What would you like to do next ?**

If you choose to save your results and quit for now (1st alternative) you will be taken to the submission page. When you are ready to continue with the survey please go back to the homepage, re-insert your contact details and choose the section you want to continue from. If you want to go ahead with the survey now, you can either choose which section to go to, or continue to the next page.
Mark only one oval.

- ◯ Quit for now, take me to the submission page *Skip to "Thank you."*
- ◯ Let me choose which section to go to *Skip to question 8.*
- ◯ Take me to the next page

M. Radiation synovectomy using 90Y, 186Re or 169Er

196. **Number of patients treated in the year 2015**

____

197. **Number of treatments performed in 2015**

____

198. **Which medical specialty owns the license to administer treatment?**

Tick all that apply.

- ☐ Nuclear Medicine
- ☐ Medical Oncology
- ☐ Radiation Oncology
- ☐ Endocrinology
- ☐ Interventional Radiology
- ☐ Other: ____________________

199. **Indications for which treatment is given**

Tick all that apply.

- ☐ Rheumatoid arthritis
- ☐ Spondylarthropathy (e.g. reactive or psoriatic arthritis)
- ☐ Other inflammatory joint diseases
- ☐ Behcet's disease
- ☐ Persistent synovial effusion
- ☐ Haemophilic arthritis
- ☐ Calcium pyrophosphate dihydrate (CPPD) arthritis
- ☐ Pigmented villonodular synovitis (PVNS)
- ☐ Persistent effusion after joint prosthesis
- ☐ Undifferentiated arthritis
- ☐ Other: ____________________

200. **Is a medical physicist involved in each treatment?**
Mark only one oval.

- Always
- Majority
- Minority
- Never

201. **Is the absorbed dose individually planned for each patient for 90Y?**
Mark only one oval.

- Always
- Majority
- Minority
- Never

202. **Is the absorbed dose individually planned for each patient for 169Er?**
Mark only one oval.

- Always
- Majority
- Minority
- Never

203. **Is the absorbed dose individually planned for each patient for 186Re?**
Mark only one oval.

- Always
- Majority
- Minority
- Never

204. **Is post-therapy imaging performed for 90Y?**
Mark only one oval.

- Always
- Majority
- Minority
- Never

205. **Is post-therapy imaging performed for 169Er?**
Mark only one oval.

- Always
- Majority
- Minority
- Never

206. **Is post-therapy imaging performed for 186Re?**
Mark only one oval.

- Always
- Majority
- Minority
- Never

207. **Is post-therapy dosimetry performed for 90Y?**
Mark only one oval.

- Always
- Majority
- Minority
- Never

208. **Is post-therapy dosimetry performed for 169Er?**
Mark only one oval.

- Always
- Majority
- Minority
- Never

209. **Is post-therapy dosimetry performed for 186Re?**
Mark only one oval.

- Always
- Majority
- Minority
- Never

210. **What basis of prescription do you typically use (specify in below box)**
Tick all that apply.

- [ ] Fixed activity
- [ ] Fixed activity based on diagnosis, stage, or other clinical factors
- [ ] Fixed activity per body surface area or patient weight
- [ ] Individually calculated activity to give a prescribed absorbed dose to target tissue
- [ ] Other prescription (specify below)

211. **Please specify the prescribed AMOUNT (90Y)**

212. **Please specify the UNIT of the prescription (90Y)**
Mark only one oval.

- MBq
- MBq / kg
- MBq / m2
- Gy

213. **Please specify the prescribed AMOUNT (169Er)**

214. **Please specify the UNIT of the prescription (169Er)**
Mark only one oval.

- MBq
- MBq / kg
- MBq / m2
- Gy

215. **Please specify the prescribed AMOUNT (186Re)**

216. **Please specify the UNIT of the prescription (186Re)**
Mark only one oval.

- MBq
- MBq / kg
- MBq / m2
- Gy

217. **Please add any additional information regarding the prescription**

218. **Is therapy repeated?**
Mark only one oval.

- Always
- Majority
- Minority
- Never

219. **If repeated therapies are performed, how many times at maximum?**
Mark only one oval.

- 1
- 2
- 3
- 4
- 5
- 6
- 7
- >7

220. **If repeated therapies are performed, which is the minimum time interval between treatments (months)?**
Mark only one oval.

- 1
- 2
- 3
- 4
- 5
- 6
- 7
- 8
- 9
- 10
- 11
- 12
- > 12

221. **What would you like to do next ?**

If you choose to save your results and quit for now (1st alternative) you will be taken to the submission page. When you are ready to continue with the survey please go back to the homepage, re-insert your contact details and choose the section you want to continue from. If you want to go ahead with the survey now, you can either choose which section to go to, or continue to the next page.
Mark only one oval.

- Quit for now, take me to the submission page *Skip to "Thank you."*
- Let me choose which section to go to *Skip to question 8.*
- Take me to the next page

N. 153Sm for bone metastases

222. **Number of patients treated in the year 2015**

223. **Number of treatments performed in 2015**

224. **Which medical specialty owns the license to administer treatment?**
Tick all that apply.

- Nuclear Medicine
- Medical Oncology
- Radiation Oncology
- Endocrinology
- Interventional Radiology
- Other:

225. **Indications (tumour type) for which treatment is given**
Tick all that apply.

- Prostate cancer bone metastases
- Breast cancer bone metastases
- Lung cancer bone metastases
- Other:

226. **Is a medical physicist involved in each treatment?**
Mark only one oval.

- Always
- Majority
- Minority
- Never

227. **Is the absorbed dose individually planned for each patient?**
Mark only one oval.

- Always
- Majority
- Minority
- Never

228. **Is post-therapy imaging performed?**
Mark only one oval.

- Always
- Majority
- Minority
- Never

229. **Is post-therapy dosimetry performed?**
Mark only one oval.

- Always
- Majority
- Minority
- Never

230. **What basis of prescription do you typically use (specify in below box)**
Tick all that apply.

- [ ] Fixed activity
- [ ] Fixed activity based on diagnosis, stage, or other clinical factors
- [ ] Fixed activity per body surface area or patient weight
- [ ] Individually calculated activity to give a prescribed absorbed dose to whole body
- [ ] Individually calculated activity to give a prescribed absorbed dose to organ at risk
- [ ] Individually calculated activity to give a prescribed absorbed dose to target tissue
- [ ] Other prescription (specify below)

231. **Please specify the prescribed AMOUNT**

____

232. **Please specify the UNIT of the prescription**
Mark only one oval.

- MBq
- MBq / kg
- MBq / m2
- Gy

233. **Please add any additional information regarding the prescription**

234. **Is therapy repeated?**
Mark only one oval.

- Always
- Majority
- Minority
- Never

235. **If repeated therapies are performed, how many times at maximum?**
Mark only one oval.

- 1
- 2
- 3
- 4
- 5
- 6
- 7
- >7

236. **If repeated therapies are performed, with which time interval between treatments (months)?**

Mark only one oval.

- 1
- 2
- 3
- 4
- 5
- 6
- 7
- 8
- 9
- 10
- 11
- 12
- > 12

237. **What would you like to do next ?**

If you choose to save your results and quit for now (1st alternative) you will be taken to the submission page. When you are ready to continue with the survey please go back to the homepage, re-insert your contact details and choose the section you want to continue from. If you want to go ahead with the survey now, you can either choose which section to go to, or continue to the next page.

Mark only one oval.

- Quit for now, take me to the submission page *Skip to "Thank you."*
- Let me choose which section to go to *Skip to question 8.*
- Take me to the next page

O. 89Sr for bone metastases

238. **Number of patients treated in the year 2015**

239. **Number of treatments performed during 2015**

240. **Which medical specialty owns the license to administer treatment?**
Tick all that apply.

- [ ] Nuclear Medicine
- [ ] Medical Oncology
- [ ] Radiation Oncology
- [ ] Endocrinology
- [ ] Interventional Radiology
- [ ] Other: ______________________

241. **Indications (tumour type) for which treatment is given**
Tick all that apply.

- [ ] Prostate cancer bone metastases
- [ ] Breast cancer bone metastases
- [ ] Lung cancer bone metastases
- [ ] Other: ______________________

242. **Is a medical physicist involved in each treatment?**
Mark only one oval.

- ( ) Always
- ( ) Majority
- ( ) Minority
- ( ) Never

243. **Is the absorbed dose individually planned for each patient?**
Mark only one oval.

- ( ) Always
- ( ) Majority
- ( ) Minority
- ( ) Never

244. **Is post-therapy imaging performed?**
Mark only one oval.

- ( ) Always
- ( ) Majority
- ( ) Minority
- ( ) Never

245. **Is post-therapy dosimetry performed?**
Mark only one oval.

- Always
- Majority
- Minority
- Never

246. **What basis of prescription do you typically use (specify in below box)**
Tick all that apply.

- Fixed activity
- Fixed activity based on diagnosis, stage, or other clinical factors
- Fixed activity per body surface area or patient weight
- Individually calculated activity to give a prescribed absorbed dose to whole body
- Individually calculated activity to give a prescribed absorbed dose to organ at risk
- Individually calculated activity to give a prescribed absorbed dose to target tissue
- Other prescription (specify below)

247. **Please specify the prescribed AMOUNT**

248. **Please specify the UNIT of the prescription**
Mark only one oval.

- MBq
- MBq / kg
- MBq / m2
- Gy

249. **Please add any additional information regarding the prescription**

250. **Is therapy repeated?**
Mark only one oval.

- Always
- Majority
- Minority
- Never

251. **If repeated therapies are performed, how many times at maximum?**
Mark only one oval.

- 1
- 2
- 3
- 4
- 5
- 6
- 7
- >7

252. **If repeated therapies are performed, with which time interval between treatments (months)?**
Mark only one oval.

- 1
- 2
- 3
- 4
- 5
- 6
- 7
- 8
- 9
- 10
- 11
- 12
- > 12

253. **What would you like to do next ?**
If you choose to save your results and quit for now (1st alternative) you will be taken to the submission page. When you are ready to continue with the survey please go back to the homepage, re-insert your contact details and choose the section you want to continue from. If you want to go ahead with the survey now, you can either choose which section to go to, or continue to the next page.
Mark only one oval.

- Quit for now, take me to the submission page *Skip to "Thank you."*
- Let me choose which section to go to *Skip to question 8.*
- Take me to the next page

P. 223Ra for bone metastases

254. **Number of patients treated in the year 2015**

____

255. **Number of treatments performed in 2015**

____

256. **Which medical specialty owns the license to administer treatment?**
Tick all that apply.

- [ ] Nuclear Medicine
- [ ] Medical Oncology
- [ ] Radiation Oncology
- [ ] Endocrinology
- [ ] Interventional Radiology
- [ ] Other: ____________________

257. **Indications (tumour type) for which treatment is given**
Tick all that apply.

- [ ] Prostate cancer bone metastases
- [ ] Breast cancer bone metastases
- [ ] Lung cancer bone metastases
- [ ] Other: ____________________

258. **Is a medical physicist involved in each treatment?**
Mark only one oval.

- ( ) Always
- ( ) Majority
- ( ) Minority
- ( ) Never

259. **Is the absorbed dose individually planned for each patient?**
Mark only one oval.

- ( ) Always
- ( ) Majority
- ( ) Minority
- ( ) Never

260. **Is post-therapy imaging performed?**
Mark only one oval.

- Always
- Majority
- Minority
- Never

261. **Is post-therapy dosimetry performed?**
Mark only one oval.

- Always
- Majority
- Minority
- Never

262. **What basis of prescription do you typically use (specify in below box)**
Tick all that apply.

- [ ] Fixed activity
- [ ] Fixed activity based on diagnosis, stage, or other clinical factors
- [ ] Fixed activity per body surface area or patient weight
- [ ] Individually calculated activity to give a prescribed absorbed dose to whole body
- [ ] Individually calculated activity to give a prescribed absorbed dose to organ at risk
- [ ] Individually calculated activity to give a prescribed absorbed dose to target tissue
- [ ] Other prescription (specify below)

263. **Please specify the prescribed AMOUNT**

____

264. **Please specify the UNIT of the prescription**
Mark only one oval.

- MBq
- MBq / kg
- MBq / m2
- Gy

265. **Please add any additional information regarding the prescription**

____
____
____
____
____

266. **Is therapy prescribed as repeated administrations?**

Mark only one oval.

- Always
- Majority
- Minority
- Never

267. **If repeated administrations are given, how many?**

Mark only one oval.

- 1
- 2
- 3
- 4
- 5
- 6
- 7
- 8
- 9
- 10
- >10

268. **If repeated administrations are given, with which time interval between them (weeks)?**

Mark only one oval.

- 1
- 2
- 3
- 4
- 5
- 6
- 7
- 8
- 9
- 10
- 11
- 12
- 13
- 14
- 15
- 16
- 17
- 18
- 19
- 20
- >20

269. **What would you like to do next ?**

If you choose to save your results and quit for now (1st alternative) you will be taken to the submission page. When you are ready to continue with the survey please go back to the homepage, re-insert your contact details and choose the section you want to continue from. If you want to go ahead with the survey now, you can either choose which section to go to, or continue to the next page.

Mark only one oval.

- Quit for now, take me to the submission page *Skip to "Thank you."*
- Let me choose which section to go to *Skip to question 8.*
- Take me to the next page

Q. 32P phosphate for myeloproliferative diseases

270. **Number of patients treated in the year 2015**

271. **Number of treatments performed in 2015**

____

272. **Which medical specialty owns the license to administer treatment?**
Tick all that apply.

- [ ] Nuclear Medicine
- [ ] Medical Oncology
- [ ] Radiation Oncology
- [ ] Endocrinology
- [ ] Interventional Radiology
- [ ] Other: ____________________

273. **Indications (tumour type) for which treatment is given**
Tick all that apply.

- [ ] Polycythaemia vera
- [ ] Essential thrombocythaemia
- [ ] Other: ____________________

274. **Is a medical physicist involved in each treatment?**
Mark only one oval.

- ( ) Always
- ( ) Majority
- ( ) Minority
- ( ) Never

275. **Is the absorbed dose individually planned for each patient?**
Mark only one oval.

- ( ) Always
- ( ) Majority
- ( ) Minority
- ( ) Never

276. **Is post-therapy imaging performed?**
Mark only one oval.

- ( ) Always
- ( ) Majority
- ( ) Minority
- ( ) Never

277. **Is post-therapy dosimetry performed?**
Mark only one oval.

- Always
- Majority
- Minority
- Never

278. **What basis of prescription do you typically use (specify in below box)**
Tick all that apply.

- Fixed activity
- Fixed activity based on diagnosis, stage, or other clinical factors
- Fixed activity per body surface area or patient weight
- Stepwise escalation of activity based on patient response
- Individually calculated activity to give a prescribed absorbed dose to whole body
- Individually calculated activity to give a prescribed absorbed dose to organ at risk
- Individually calculated activity to give a prescribed absorbed dose to target tissue
- Other prescription (specify below)

279. **Please specify the prescribed AMOUNT**

280. **Please specify the UNIT of the prescription**
Mark only one oval.

- MBq
- MBq / kg
- MBq / m2
- Gy

281. **Please add any additional information regarding the prescription**

282. **Is therapy repeated?**
Mark only one oval.

- Always
- Majority
- Minority
- Never

283. **If repeated therapies are performed, how many times at maximum?**
Mark only one oval.

- 1
- 2
- 3
- 4
- 5
- 6
- 7
- 8
- 9
- 10
- 11
- 12
- >12

284. **If repeated therapies are performed, with which time interval between treatments (months)?**
Mark only one oval.

- 1
- 2
- 3
- 4
- 5
- 6
- 7
- 8
- 9
- 10
- 11
- 12
- > 12

285. **What would you like to do next ?**

If you choose to save your results and quit for now (1st alternative) you will be taken to the submission page. When you are ready to continue with the survey please go back to the homepage, re-insert your contact details and choose the section you want to continue from. If you want to go ahead with the survey now, you can either choose which section to go to, or continue to the next page.
Mark only one oval.

- Quit for now, take me to the submission page *Skip to "Thank you."*
- Let me choose which section to go to *Skip to question 8.*
- Take me to the next page

R. 90Y Zevalin for B-cell lymphoma

286. **Number of patients treated in the year 2015**

287. **Number of treatments performed in 2015**

288. **Which medical specialty owns the license to administer treatment?**

Tick all that apply.

- [ ] Nuclear Medicine
- [ ] Medical Oncology
- [ ] Radiation Oncology
- [ ] Endocrinology
- [ ] Interventional Radiology
- [ ] Other:

289. **Is a medical physicist involved in each treatment?**

Mark only one oval.

- Always
- Majority
- Minority
- Never

290. **Is the absorbed dose individually planned for each patient?**

Mark only one oval.

- Always
- Majority
- Minority
- Never

291. **Is post-therapy imaging performed?**
Mark only one oval.

- Always
- Majority
- Minority
- Never

292. **Is post-therapy dosimetry performed?**
Mark only one oval.

- Always
- Majority
- Minority
- Never

293. **What basis of prescription do you typically use (specify in below box)**
Tick all that apply.

- Fixed activity
- Fixed activity based on diagnosis, stage, or other clinical factors
- Fixed activity per body surface area or patient weight
- Stepwise escalation of activity based on patient response
- Individually calculated activity to give a prescribed absorbed dose to whole body
- Individually calculated activity to give a prescribed absorbed dose to organ at risk
- Individually calculated activity to give a prescribed absorbed dose to target tissue
- Other prescription (specify below)

294. **Please specify the prescribed AMOUNT**

295. **Please specify the UNIT of the prescription**
Mark only one oval.

- MBq
- MBq / kg
- MBq /m2
- Gy

296. **Please add any additional information regarding the prescription**

297. **What would you like to do next ?**

If you choose to save your results and quit for now (1st alternative) you will be taken to the submission page. When you are ready to continue with the survey please go back to the homepage, re-insert your contact details and choose the section you want to continue from. If you want to go ahead with the survey now, you can either choose which section to go to, or continue to the next page.

Mark only one oval.

- Quit for now, take me to the submission page *Skip to "Thank you."*
- Let me choose which section to go to *Skip to question 8.*
- Take me to the next page

S. Therapy using alpha-emitting radionuclides other than 223Ra (option 1)

This question is repeated three times, in case you are applying several therapies with alpha-emitting radionuclides. Please fill in each therapy separately.

298. **Which radionuclide is used?**

299. **Which targeting molecule is used?**

300. **Which disease is being treated?**

301. **Number of patients treated per year**

302. **Number of treatments performed per year**

303. **Which medical specialty owns the license to administer treatment?**
Tick all that apply.

- [ ] Nuclear Medicine
- [ ] Medical Oncology
- [ ] Radiation Oncology
- [ ] Endocrinology
- [ ] Interventional Radiology
- [ ] Other: ______________________

304. **Is a medical physicist involved in each treatment?**
Mark only one oval.

- Always
- Majority
- Minority
- Never

305. **Is the absorbed dose individually planned for each patient?**
Mark only one oval.

- Always
- Majority
- Minority
- Never

306. **Is post-therapy imaging performed?**
Mark only one oval.

- Always
- Majority
- Minority
- Never

307. **Is post-therapy dosimetry performed?**
Mark only one oval.

- Always
- Majority
- Minority
- Never

308. **What basis of prescription do you typically use (specify in below box)**
Tick all that apply.

- [ ] Fixed activity
- [ ] Fixed activity based on diagnosis, stage, or other clinical factors
- [ ] Fixed activity per body surface area or patient weight
- [ ] Individually calculated activity to give a prescribed absorbed dose to whole body
- [ ] Individually calculated activity to give a prescribed absorbed dose to organ at risk
- [ ] Individually calculated activity to give a prescribed absorbed dose to target tissue
- [ ] Other prescription (specify below)

309. **Please specify the prescribed AMOUNT**

____

310. **Please specify the UNIT of the prescription**
Mark only one oval.

- ( ) MBq
- ( ) MBq / kg
- ( ) MBq / m2
- ( ) Gy

311. **Please add any additional information regarding the prescription**

____
____
____
____
____

312. **What would you like to do next ?**
If you choose to save your results and quit for now (1st alternative) you will be taken to the submission page. When you are ready to continue with the survey please go back to the homepage, re-insert your contact details and choose the section you want to continue from. If you want to go ahead with the survey now, you can either choose which section to go to, or continue to the next page.
Mark only one oval.

- ( ) Quit for now, take me to the submission page *Skip to "Thank you."*
- ( ) Let me choose which section to go to *Skip to question 8.*
- ( ) Take me to the next page

T. Therapy using alpha-emitting radionuclides other than 223Ra (option 2)

This question is repeated three times, in case you are applying several therapies with alpha-emitting radionuclides. Please fill in each therapy separately.

313. **Which radionuclide is used?**

314. **Which targeting molecule is used?**

315. **Which disease is being treated?**

316. **Number of patients treated per year**

317. **Number of treatments performed per year**

318. **Which medical specialty owns the license to administer treatment?**
Tick all that apply.

- [ ] Nuclear Medicine
- [ ] Medical Oncology
- [ ] Radiation Oncology
- [ ] Endocrinology
- [ ] Interventional Radiology
- [ ] Other: ____________

319. **Is a medical physicist involved in each treatment?**
Mark only one oval.

- ( ) Always
- ( ) Majority
- ( ) Minority
- ( ) Never

320. **Is the absorbed dose individually planned for each patient?**
Mark only one oval.

- ( ) Always
- ( ) Majority
- ( ) Minority
- ( ) Never

321. **Is post-therapy imaging performed?**
Mark only one oval.

- Always
- Majority
- Minority
- Never

322. **Is post-therapy dosimetry performed?**
Mark only one oval.

- Always
- Majority
- Minority
- Never

323. **What basis of prescription do you typically use (specify in below box)**
Tick all that apply.

- [ ] Fixed activity
- [ ] Fixed activity based on diagnosis, stage, or other clinical factors
- [ ] Fixed activity per body surface area or patient weight
- [ ] Individually calculated activity to give a prescribed absorbed dose to whole body
- [ ] Individually calculated activity to give a prescribed absorbed dose to organ at risk
- [ ] Individually calculated activity to give a prescribed absorbed dose to target tissue
- [ ] Other prescription (specify below)

324. **Please specify the prescribed AMOUNT**

____

325. **Please specify the UNIT of the prescription**
Mark only one oval.

- MBq
- MBq / kg
- MBq / m2
- Gy

326. **Please add any additional information regarding the prescription**

____
____
____
____
____

327. **What would you like to do next ?**

If you choose to save your results and quit for now (1st alternative) you will be taken to the submission page. When you are ready to continue with the survey please go back to the homepage, re-insert your contact details and choose the section you want to continue from. If you want to go ahead with the survey now, you can either choose which section to go to, or continue to the next page.
Mark only one oval.

- Quit for now, take me to the submission page *Skip to "Thank you."*
- Let me choose which section to go to *Skip to question 8.*
- Take me to the next page

U. Therapy using alpha-emitting radionuclides other than 223Ra (option 3)

This question is repeated three times, in case you are applying several therapies with alpha-emitting radionuclides. Please fill in each therapy separately.

328. **Which radionuclide is used?**

____

329. **Which targeting molecule is used?**

____

330. **Which disease is being treated?**

____

331. **Number of treatments performed per year**

____

332. **Number of patients treated per year**

____

333. **Which medical specialty owns the license to administer treatment?**

Tick all that apply.

- [ ] Nuclear Medicine
- [ ] Medical Oncology
- [ ] Radiation Oncology
- [ ] Endocrinology
- [ ] Interventional Radiology
- [ ] Other: ______________________

334. **Is a medical physicist involved in each treatment?**
Mark only one oval.

- Always
- Majority
- Minority
- Never

335. **Is the absorbed dose individually planned for each patient?**
Mark only one oval.

- Always
- Majority
- Minority
- Never

336. **Is post-therapy imaging performed?**
Mark only one oval.

- Always
- Majority
- Minority
- Never

337. **Is post-therapy dosimetry performed?**
Mark only one oval.

- Always
- Majority
- Minority
- Never

338. **What basis of prescription do you typically use (specify in below box)**
Tick all that apply.

- [ ] Fixed activity
- [ ] Fixed activity based on diagnosis, stage, or other clinical factors
- [ ] Fixed activity per body surface area or patient weight
- [ ] Individually calculated activity to give a prescribed absorbed dose to whole body
- [ ] Individually calculated activity to give a prescribed absorbed dose to organ at risk
- [ ] Individually calculated activity to give a prescribed absorbed dose to target tissue
- [ ] Other prescription (specify below)

339. **Please specify the prescribed AMOUNT**

340. **Please specify the UNIT of the prescription**
Mark only one oval.

- MBq
- MBq / kg
- MBq / m2
- Gy

341. **Please add any additional information regarding the prescription**

342. **What would you like to do next ?**
If you choose to save your results and quit for now (1st alternative) you will be taken to the submission page. When you are ready to continue with the survey please go back to the homepage, re-insert your contact details and choose the section you want to continue from. If you want to go ahead with the survey now, you can either choose which section to go to, or continue to the next page.
Mark only one oval.

- Quit for now, take me to the submission page *Skip to "Thank you."*
- Let me choose which section to go to *Skip to question 8.*
- Take me to the next page

V. Therapy using other radiopharmaceutical (option 1)

This question is repeated three times, in case you are applying several therapies with other, not listed, radiopharmaceuticals. Please fill in each therapy separately.

343. **Which radionuclide is used?**

344. **Which targeting molecule is used?**

345. **Which disease is being treated?**

346. **Number of patients treated per year**

347. **Number of treatments performed per year**

348. **Which medical specialty owns the license to administer treatment?**
Tick all that apply.

- [ ] Nuclear Medicine
- [ ] Medical Oncology
- [ ] Radiation Oncology
- [ ] Endocrinology
- [ ] Interventional Radiology
- [ ] Other: ____________________

349. **Is a medical physicist involved in each treatment?**
Mark only one oval.

- Always
- Majority
- Minority
- Never

350. **Is the absorbed dose individually planned for each patient?**
Mark only one oval.

- Always
- Majority
- Minority
- Never

351. **Is post-therapy imaging performed?**
Mark only one oval.

- Always
- Majority
- Minority
- Never

352. **Is post-therapy dosimetry performed?**
Mark only one oval.

- Always
- Majority
- Minority
- Never

353. **What basis of prescription do you typically use (specify in below box)**
Tick all that apply.

- [ ] Fixed activity
- [ ] Fixed activity based on diagnosis, stage, or other clinical factors
- [ ] Fixed activity per body surface area or patient weight
- [ ] Individually calculated activity to give a prescribed absorbed dose to whole body
- [ ] Individually calculated activity to give a prescribed absorbed dose to organ at risk
- [ ] Individually calculated activity to give a prescribed absorbed dose to target tissue
- [ ] Other prescription (specify below)

354. **Please specify the prescribed AMOUNT**

____

355. **Please specify the UNIT of the prescription**
Mark only one oval.

- ( ) MBq
- ( ) MBq / kg
- ( ) MBq / m2
- ( ) Gy

356. **Please add any additional information regarding the prescription**

____
____
____
____
____

357. **What would you like to do next ?**
If you choose to save your results and quit for now (1st alternative) you will be taken to the submission page. When you are ready to continue with the survey please go back to the homepage, re-insert your contact details and choose the section you want to continue from. If you want to go ahead with the survey now, you can either choose which section to go to, or continue to the next page.
Mark only one oval.

- ( ) Quit for now, take me to the submission page *Skip to "Thank you."*
- ( ) Let me choose which section to go to *Skip to question 8.*
- ( ) Take me to the next page

W. Therapy using other radiopharmaceutical (option 2)

This question is repeated three times, in case you are applying several therapies with other, not listed, radiopharmaceuticals. Please fill in each therapy separately.

358. **Which radionuclide is used?**

359. **Which targeting molecule is used?**

360. **Which disease is being treated?**

361. **Number of patients treated per year**

362. **Number of treatments performed per year**

363. **Which medical specialty owns the license to administer treatment?**
Tick all that apply.

- [ ] Nuclear Medicine
- [ ] Medical Oncology
- [ ] Radiation Oncology
- [ ] Endocrinology
- [ ] Interventional Radiology
- [ ] Other:

364. **Is a medical physicist involved in each treatment?**
Mark only one oval.

- Always
- Majority
- Minority
- Never

365. **Is the absorbed dose individually planned for each patient?**
Mark only one oval.

- Always
- Majority
- Minority
- Never

366. **Is post-therapy imaging performed?**
Mark only one oval.

- Always
- Majority
- Minority
- Never

367. **Is post-therapy dosimetry performed?**
Mark only one oval.

- Always
- Majority
- Minority
- Never

368. **What basis of prescription do you typically use (specify in below box)**
Tick all that apply.

- [ ] Fixed activity
- [ ] Fixed activity based on diagnosis, stage, or other clinical factors
- [ ] Fixed activity per body surface area or patient weight
- [ ] Individually calculated activity to give a prescribed absorbed dose to whole body
- [ ] Individually calculated activity to give a prescribed absorbed dose to organ at risk
- [ ] Individually calculated activity to give a prescribed absorbed dose to target tissue
- [ ] Other prescription (specify below)

369. **Please specify the prescribed AMOUNT**

____

370. **Please specify the UNIT of the prescription**
Mark only one oval.

- MBq
- MBq / kg
- MBq / m2
- Gy

371. **Please add any additional information regarding the prescription**

____
____
____
____
____

372. **What would you like to do next ?**

If you choose to save your results and quit for now (1st alternative) you will be taken to the submission page. When you are ready to continue with the survey please go back to the homepage, re-insert your contact details and choose the section you want to continue from. If you want to go ahead with the survey now, you can either choose which section to go to, or continue to the next page.

Mark only one oval.

- ◯ Quit for now, take me to the submission page *Skip to "Thank you."*
- ◯ Let me choose which section to go to *Skip to question 8.*
- ◯ Take me to the next page

X. Therapy using other radiopharmaceutical (option 3)

This question is repeated three times, in case you are applying several therapies with other, not listed, radiopharmaceuticals. Please fill in each therapy separately.

373. **Which radionuclide is used?**

____

374. **Which targeting molecule is used?**

____

375. **Which disease is being treated?**

____

376. **Number of patients treated per year**

____

377. **Number of treatments performed per year**

____

378. **Which medical specialty owns the license to administer treatment?**

Tick all that apply.

- ☐ Nuclear Medicine
- ☐ Medical Oncology
- ☐ Radiation Oncology
- ☐ Endocrinology
- ☐ Interventional Radiology
- ☐ Other: ____________________________

379. **Is a medical physicist involved in each treatment?**
Mark only one oval.

- Always
- Majority
- Minority
- Never

380. **Is the absorbed dose individually planned for each patient?**
Mark only one oval.

- Always
- Majority
- Minority
- Never

381. **Is post-therapy imaging performed?**
Mark only one oval.

- Always
- Majority
- Minority
- Never

382. **Is post-therapy dosimetry performed?**
Mark only one oval.

- Always
- Majority
- Minority
- Never

383. **What basis of prescription do you typically use (specify in below box)**
Tick all that apply.

- [ ] Fixed activity
- [ ] Fixed activity based on diagnosis, stage, or other clinical factors
- [ ] Fixed activity per body surface area or patient weight
- [ ] Individually calculated activity to give a prescribed absorbed dose to whole body
- [ ] Individually calculated activity to give a prescribed absorbed dose to organ at risk
- [ ] Individually calculated activity to give a prescribed absorbed dose to target tissue
- [ ] Other prescription (specify below)

384. **Please specify the prescribed AMOUNT**

385. **Please specify the UNIT of the prescription**
Mark only one oval.

- MBq
- MBq / kg
- MBq / m2
- Gy

386. **Please add any additional information regarding the prescription**

387. **What would you like to do next ?**
If you choose to save your results and quit for now (1st alternative) you will be taken to the submission page. When you are ready to continue with the survey please go back to the homepage, re-insert your contact details and choose the section you want to continue from. If you want to go ahead with the survey now, you can either choose which section to go to, or continue to the next page.
Mark only one oval.

- Quit for now, take me to the submission page *Skip to "Thank you."*
- Let me choose which section to go to *Skip to question 8.*
- Take me to the next page

Y. General questions

388. **Are you satisfied with the current implementation of patient-specific dosimetry in molecular radiotherapy in your centre?**
Mark only one oval.

- YES
- NO

389. **In general, patient-specific dosimetry requires a number of scintigraphic scans and staff resources. If you answered NO above: Which factors limit the implementation of patient-specific dosimetry in your center?**

Tick all that apply.

- [ ] Shortage of knowledge and know-how
- [ ] Shortage of medical physicists working in nuclear medicine
- [ ] Shortage of other staff
- [ ] Limited access to scanner or other equipment needed
- [ ] Limited access to dedicated software
- [ ] No legislative requirement to perform dosimetry
- [ ] Other (specify below)

390. **If you answered OTHER to the question above, please specify**

391. **Do you perform research in molecular radiotherapy?**

Mark only one oval.

- ( ) YES (please specify briefly in below box)
- ( ) NO

392. **If you answered YES to the question above: please specify which research you carry out in molecular radiotherapy at your centre**

393. **Do you have any other comments? (200 words maximum)**

394. **What would you like to do next ?**

If you choose to save your results and quit for now (1st alternative) you will be taken to the submission page. When you are ready to continue with the survey please go back to the homepage, re-insert your contact details and choose the section you want to continue from. If you want to go ahead with the survey now, you can either choose which section to go to, or continue to the next page.

Mark only one oval.

- Quit for now, take me to the submission page *Skip to "Thank you."*
- Let me choose which section to go to *Skip to question 8.*
- Take me to the next page

Skip to "Thank you."

Thank you

Thank you for your responses, and the time and effort you devoted to filling in this survey. Click the Submit Button.

EANM Internal Dosimetry Task Force

Powered by
Google Forms
